# Macrophage deficiency of Akt2 reduces atherosclerosis in *Ldlr* null mice[S]

**DOI:** 10.1194/jlr.M050633

**Published:** 2014-11

**Authors:** Vladimir R. Babaev, Katie E. Hebron, Carrie B. Wiese, Cynthia L. Toth, Lei Ding, Youmin Zhang, James M. May, Sergio Fazio, Kasey C. Vickers, MacRae F. Linton

**Affiliations:** * Atherosclerosis Research Unit, Department of Medicine, Vanderbilt University Medical Center, Nashville, TN 37232; ** Department of Pathology, Vanderbilt University Medical Center, Nashville, TN 37232; § Department of Molecular Physiology and Biophysics, Vanderbilt University Medical Center, Nashville, TN 37232; † Department of Pharmacology, Vanderbilt University Medical Center, Nashville, TN 37232

**Keywords:** macrophages/monocytes, cell signaling, apoptosis, foam cells

## Abstract

Macrophages play crucial roles in the formation of atherosclerotic lesions. Akt, a serine/threonine protein kinase B, is vital for cell proliferation, migration, and survival. Macrophages express three Akt isoforms, Akt1, Akt2, and Akt3, but the roles of Akt1 and Akt2 in atherosclerosis in vivo remain unclear. To dissect the impact of macrophage Akt1 and Akt2 on early atherosclerosis, we generated mice with hematopoietic deficiency of Akt1 or Akt2. After 8 weeks on Western diet, *Ldlr*^−/−^ mice reconstituted with *Akt1*^−/−^ fetal liver cells (*Akt1*^−/−^→*Ldlr*^−/−^) had similar atherosclerotic lesion areas compared with control mice transplanted with WT cells (WT→*Ldlr*^−/−^). In contrast, *Akt2*^−/−^→*Ldlr*^−/−^ mice had dramatically reduced atherosclerotic lesions compared with WT→*Ldlr*^−/−^ mice of both genders. Similarly, in the setting of advanced atherosclerotic lesions, Akt2^−/−^→*Ldlr*^−/−^ mice had smaller aortic lesions compared with WT→*Ldlr*^−/−^ and *Akt1*^−/−^→*Ldlr*^−/−^ mice. Importantly, *Akt2*^−/−^→*Ldlr*^−/−^ mice had reduced numbers of proinflammatory blood monocytes expressing Ly-6C^hi^ and chemokine C-C motif receptor 2. Peritoneal macrophages isolated from *Akt2*^−/−^ mice were skewed toward an M2 phenotype and showed decreased expression of proinflammatory genes and reduced cell migration. Our data demonstrate that loss of Akt2 suppresses the ability of macrophages to undergo M1 polarization reducing both early and advanced atherosclerosis.

Macrophages play key roles in atherogenesis, and the quantity and phenotype of these cells in atherosclerotic lesions influence both disease progression and regression (1). Akt, a serine/threonine protein kinase B, is an important signaling mediator that regulates a wide variety of cellular functions, including metabolism, migration, and cell survival (2). In mouse macrophages, Akt signaling is constitutively active and essential for their survival (3). Macrophages express three Akt isoforms, Akt1, Akt2, and Akt3, which are products of distinct genes exhibiting high sequence homology (2). These isoforms may have isoform-specific or redundant effects on Akt signaling, depending on the cell type and conditions. Several recent studies have used Akt isoform knockout approaches to demonstrate isoform-specific functions. For example, mice lacking Akt1 (*Akt*1^−/−^) have increased perinatal mortality and reduced body weight (4, 5). In contrast, *Akt*2^−/−^ mice display normal growth but exhibit a diabetes-like syndrome (6), whereas Akt3^−/−^ mice exhibit a reduction in brain volume (7). With regard to vascular effects, Akt1 expression by endothelial cells mediates protective and prosurvival effects (8). Fernández-Hernando et al. (9) reported that the loss of *Akt1* in apoE-deficient (*Apoe*^−/−^) mice leads to severe atherosclerosis largely due to impaired endothelial cell function. In addition, the loss of *Akt1* reduces vascular smooth muscle cell (VSMC) migration and survival (10). Similarly, *Akt3* deficiency in macrophages of *Apoe*^−/−^ mice promoted foam cell formation and atherosclerosis (11). Recently, double knockout (DKO) mice for *Akt2* and *Ldlr* have been reported to develop impaired glucose tolerance and more complex atherosclerotic plaques compared with *Ldlr*^−/−^ controls (12). The impact on lesion composition was attributed, at least in part, to VSMC migration, proliferation, and elaboration of collagen and matrix metalloproteinases (12). However, the impact of macrophage phenotypes generated by *Akt1* and *Akt2* deficiency on atherosclerosis remains unclear.

Depending on environmental signals, macrophages can acquire two distinct functional phenotypes. Classically activated M1 macrophages are defined by their response to IFNγ or lipopolysaccharide (LPS), whereas alternatively activated M2 macrophages are defined by responses to interleukin (IL) 4 or IL-13 (13, 14). The M1 phenotype is proinflammatory and is characterized by increased release of cytokines and reactive oxygen intermediates. In contrast, M2 macrophages show an immunosuppressive phenotype with an enhanced production of anti-inflammatory cytokines and substantial scavenging activity. These macrophage phenotypes can be reversibly shifted in response to changes in the cytokine environment (15). The M1 macrophages are thought to play important roles in plaque initiation, progression, and instability (16, 17); whereas the M2 phenotype has been implicated in resolution of inflammation, plaque stability, and regression of atherosclerosis (18). Priming macrophages to the M1 or M2 phenotype influences their inflammatory potential (13) and, therefore, may impact the development of atherosclerosis (16, 17).

Initial studies have shown that the phosphatidylinositol 3-kinase (PI3K)/Akt pathway is important for macrophage polarization (19) and migration (20). A recent report by Arranz et al. (21) demonstrated that Akt kinase differentially contributes to macrophage polarization, with Akt1 ablation giving rise to M1, and Akt2 deletion promoting the M2 phenotype in an in vivo model of colitis in mice. Changes in macrophage polarization may dramatically affect atherogenesis (16, 22). However, the impact of altered macrophage polarization to M1 and M2 phenotypes caused by *Akt1* or *Akt2* deficiency, respectively, on atherogenesis remains unknown.

Here we used a genetic loss-of-function approach to investigate the impact of deficiency of *Akt1* and *Akt2* in hematopoietic cells on atherosclerosis in *Ldlr*^−/−^ mice. Our data support a critical role for Akt2 in macrophage polarization, which modulates the development of both early and advanced atherosclerotic lesions.

## MATERIALS AND METHODS

### Animal procedures

Mice with knockouts for the *Akt1* (5) and *Akt2* (6, 23) genes were on the C57BL/6J background (10th backcross) and purchased from the Jackson Laboratory. The recipient *Ldlr*^−/−^ mice were also on the C57BL/6 background from the Jackson Laboratory. Mice were maintained in microisolator cages on a rodent chow diet containing 4.5% fat (PMI 5010, St. Louis, MO) or a Western type diet containing 21% milk fat and 0.15% cholesterol (Teklad, Madison, WI). Animal care and experimental procedures were performed according to the regulations of Vanderbilt University’s Institutional Animal Care and Usage Committee.

### Fetal liver cell transplantation

Fetal liver cells (FLCs) were isolated on days 14 to 16 of gestation. Genotypes (using protocols of the Jackson Laboratory) and the gender of the embryos (24) were determined by PCR. Recipient *Ldlr*^−/−^ or C57BL/6 mice were lethally irradiated (9 Gy) and transplanted with FLCs (4 × 10^6^).

### Analysis of serum lipids and aortic lesions

Serum total cholesterol and triglyceride levels were determined on overnight fasting samples. Aortas were flushed through the left ventricle, and the entire aorta was dissected for en face analysis (25). Cryosections of the proximal aorta were stained by Oil Red O and analyzed using an Imaging system KS 300 (Kontron Electronik GmbH¸ Duesseldorf, Germany) as described (25).

### Immunocytochemistry

To detect macrophages, 5 μm cryosections of the proximal aorta were fixed in acetone at 4°C and treated with rat monoclonal antibodies to mouse macrophages, MOMA-2, or CD68 (both from AbD Serotec, Raleigh, NC). The sections were treated with goat biotinylated antibodies to chicken IgG (Vector Laboratories, Burlingame, CA) or to rat IgG (PharMingen, San Diego, CA) for 45 min at 37°C and incubated with avidin-biotin complex labeled with alkaline phosphatase (Vector Laboratories). Enzyme was viewed with Fast Red TR/Naphthol AS-NX substrate (Sigma-Aldrich Corporation, St. Louis, MO). Nonimmune rat serum was used in the place of primary antibody as a negative control. Photomicroscopy was performed on an Olympus AX70 microscope with a DP72 camera (Center Valley, PA).

### Peritoneal macrophage isolation

Thioglycollate-elicited mouse peritoneal macrophages were isolated and, 2 days later, incubated with serum-free media overnight. Macrophages were treated with insulin (Sigma) or recombinant mouse epidermal growth factor (R & D Systems, Minneapolis, MN).

### Western blotting

Cells were lysed in a lysis buffer [Cell Signaling Technology (CST), Danvers, MA] containing protease and phosphatase inhibitors. Proteins were measured with the DC Protein assay kit (Bio-Rad Laboratories, Hercules, CA) and resolved by NuPAGE Bis-Tris electrophoresis and transferred onto nitrocellulose membranes (Amersham Bioscience, Piscataway, NJ). Blots were probed with rabbit antibodies to Akt, Akt1, Akt2 or pan-Akt, p-Akt S^473^ (all from CST), or β-actin antibodies (Abcam Inc., Cambridge, MA) and goat anti-rabbit horseradish peroxidase-conjugated secondary antibodies (Sigma). Proteins were visualized with ECL Western blotting detection reagents (GE Healthcare, Piscataway, NJ) and quantified by densitometry using ImageJ software (National Institutes of Health).

### RNA isolation and real-time PCR

Total RNA was isolated and relative quantitation of the target mRNA was performed as described (25). The gene expression assays (Applied Biosystems, Foster City, CA) were normalized with 18S rRNA as an endogenous control.

### Flow cytometry

Analysis of the blood cell surface markers was performed in the Research Flow Cytometry Core Laboratory, Veterans Administration Medical Center using FACS DiVa v6.1 software (BD Biosciences) and a set of antibodies including antibodies to mouse CD11b, CD115, Ly-6C, Ly-6G (all from BioLegend), and mouse chemokine C-C motif receptor 2 (CCR2; R and D Systems).

### Priming macrophages to M1 and M2 phenotypes

Peritoneal macrophages were cultured in DMEM media containing 10% FBS only (control) or with recombinant mouse IFNγ (50 ng/ml, EMD Millipore, Billerica, MA) or with recombinant mouse IL-4 (20 ng/ml, Thermo Scientific, Rockford, IL) for 24 or 48 h. Proteins were resolved by electrophoresis (50 μg/well) and analyzed by Western blot using antibodies to CCR2 (Epitomics, Burlingame, CA), Ym1 (StemCell Technologies, Vancouver, Canada), or arginase 1 (Arg1; BD Bioscience, San Jose, CA) and β-actin (Abcam).

### Macrophage (Boyden) migration assay

WT, *Akt1*^−/−^, and *Akt2*^−/−^ peritoneal macrophages were cultured at 37°C for 2–3 h in FluoroBlok Transwell inserts (3 μm pores, BD Bioscience) that block light transmission, simplifying detection of migrating cells, and then monocyte chemoattractant protein-1 (MCP-1; 100 ng/ml, R and D Systems) was added into the lower chamber, and cells were incubated for 2 h at 37°C. The membranes were stained with 4,6-diamidino-2-phenylindole (DAPI) and analyzed under a fluorescent microscope.

### Apoptosis assessment

Cryosections of 5 μm were fixed in 4% paraformaldehyde in PBS, and apoptotic cells were detected by the in situ cell death detection kit (Roche Applied Science, Indianapolis, IN) as described (25). Terminal deoxynucleotidyl transferase-mediated dUTP nick-end-labeling (TUNEL)-positive (TUNEL^+^) cells were counted in four different sections of each aorta.

### microRNA analysis

Total RNA was isolated from mouse peritoneal macrophages using Norgen Total RNA Isolation Kits, as per the manufacturer’s instructions. Total RNA (225 ng) was reverse transcribed using TaqMan microRNA RT Kit with Rodent RT Pools A and B (v3.0) as per protocol (Life Technologies). microRNA (miRNA) cDNA was amplified using MegaPlex PreAmp Primer Rodent Pools A and B (v3.0) with PreAmp Master Mix for 12 cycles (Life Technologies), and product was diluted 1:40 and spotted with TaqMan Universal PCR Master Mix onto Rodent MicroRNA OpenArrays using an AccuFill system. OpenArrays real-time PCR-based analysis of mouse miRNAs was completed using the QuantStudio 12f-flex system, and data were analyzed as relative quantitative values normalized to U6 housekeeping RNA levels. High-level miRNA analysis was completed using GeneSpring GX_12.6 and generic import templates.

### Statistical analysis

The statistical differences in mean serum lipids and aortic lesion areas between the groups were determined by SigmaStat V.2 (SPSS Inc.).

## RESULTS

### Deficiency of Akt2 in hematopoietic cells suppresses early atherosclerosis

**Fig. 1.**
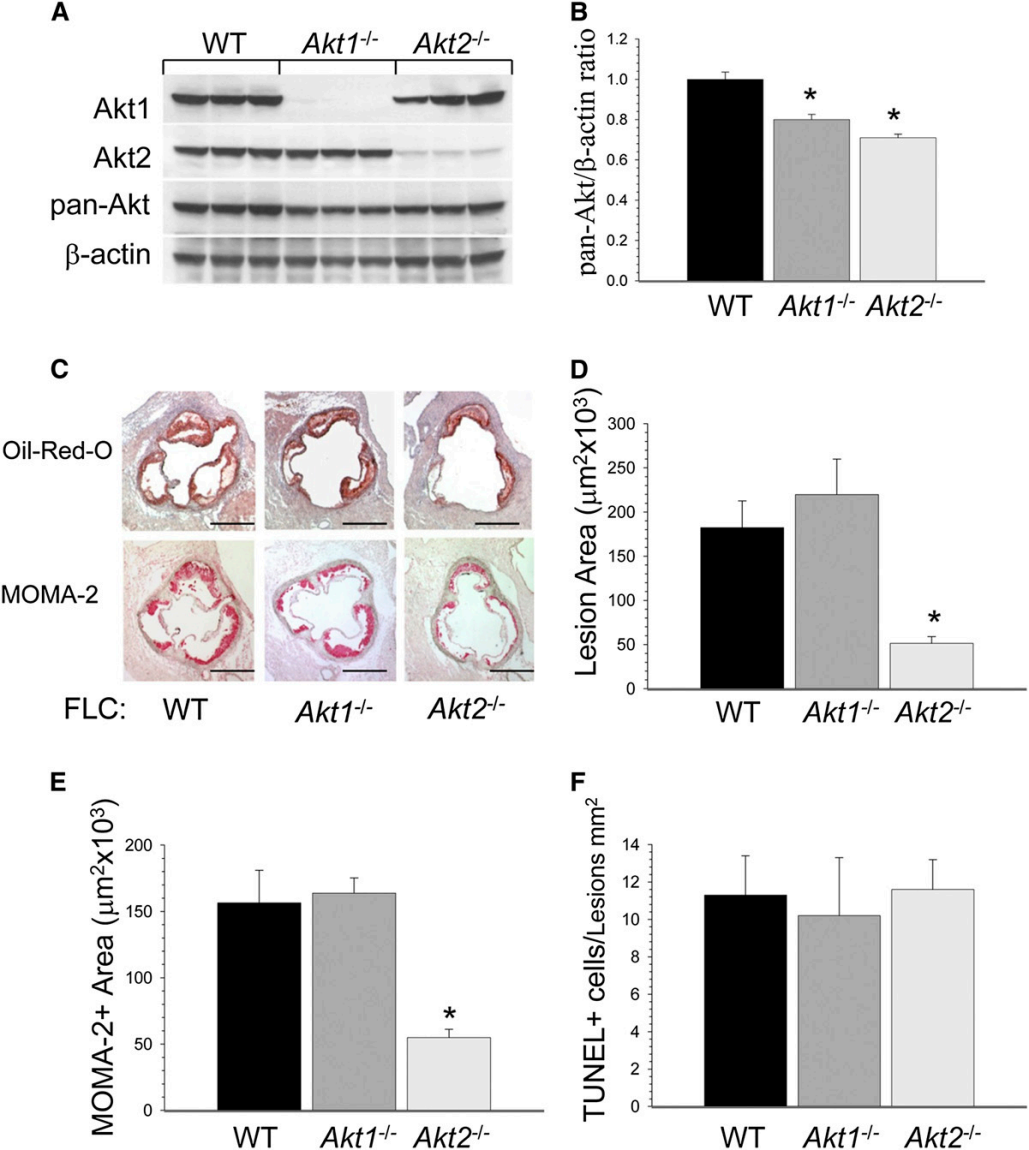
Loss of *Akt1* and *Akt2* decreases Akt levels in macrophages, but only Akt2 deficiency in hematopoietic cells reduces early atherosclerosis in female *Ldlr*^−/−^ mice. A, B: Macrophage *Akt1* and *Akt2* deficiency significantly reduces total Akt protein levels in the proportion of WT > *Akt1*^−/−^ > *Akt2*^−/−^ (* *P <* 0.05 by one-way ANOVA, multiple comparison procedures, Holm-Sidak method). Peritoneal macrophages were isolated from *Ldlr*^−/−^ mice reconstituted with WT (black), *Akt1*^−/−^ (dark gray), and *Akt2*^−/−^ (light gray) FLCs (n = 3/group), then proteins were extracted, resolved (50 μg/well) and analyzed by Western blot using an Akt isoform sampler kit. The graph (B) presents an average (mean ± SEM) of the ratio of pan-Akt/β-actin in macrophages. C: *Akt2*^−/−^→*Ldlr*^−/−^ mice had significantly smaller atherosclerotic lesions in the aortic sinus than WT→*Ldlr*^−/−^ or *Akt1*^−/−^→*Ldlr*^−/−^ mice after 8 weeks on the Western diet. Serial cryostat sections were stained with Oil Red O to detect neutral lipids or with MOMA-2 antibody to reveal macrophages. Scale bars, 200 μm. D–F: The extent of atherosclerotic lesions in the aortic sinus (D), MOMA-2-positive area (E), and apoptotic cell numbers (F) in the aortic lesions of *Ldlr*^−/−^ mice reconstituted with WT (black), *Akt1*^−/−^(dark gray), or *Akt2*^−/−^ (light gray) FLCs (* *P <* 0.05 vs. control WT→*Ldlr*^−/−^ mice by a one-way ANOVA on ranks, Tukey test).

Because *Akt1* knockout mice exhibit lower fertility and high prenatal mortality (5), we utilized the FLC transplantation approach (24) to generate mice with hematopoietic deficiency of Akt1 or Akt2. To dissect the roles of macrophage Akt1 and Akt2 isoforms in atherosclerosis, 10-week-old female *Ldlr*^−/−^ mice were lethally irradiated and transplanted with female WT (n = 9), *Akt1*^−/−^ (n = 10), or *Akt*2^−/−^ (n = 10) FLCs. Four weeks posttransplantation, the recipient mice were placed on a Western diet for 8 weeks. As expected, peritoneal macrophages isolated from *Akt1*^−/−^→*Ldlr*^−/−^ or *Akt2*^−/−^→*Ldlr*^−/−^ recipients had virtually no expression of Akt1 or Akt2 proteins, respectively (**Fig. 1A**). Total Akt protein levels were significantly decreased in both knockout cell types in the following proportion WT > *Akt1*^−/−^ > *Akt2*^−/−^ (Fig. 1B).

At euthanization, the recipient mice reconstituted with WT, *Akt*^−/−^, or *Akt2*^−/−^ FLCs did not differ by body weight, serum total cholesterol levels (**Table 1**), or lipoprotein distributions (data not shown). *Akt1*^−/−^→*Ldlr*^−/−^ mice had similarly sized atherosclerotic lesions in cross-sections of the proximal aorta compared with control WT→*Ldlr*^−/−^ mice (Fig. 1C, D). In contrast, *Akt2*^−/−^→*Ldlr*^−/−^ mice developed significantly smaller (by 71%) atherosclerotic lesions in the aortic sinus than mice transplanted with either WT or *Akt1*^−/−^ FLCs (Fig. 1C, D; 51.3 ± 7.8 vs. 182.3 ± 30.3 and 219.7 ± 40.2 × 10^3^ μm^2^). In addition, *Akt2*^−/−^→Ldlr^−/−^ mice had significantly less macrophage staining area in atherosclerotic lesions than *Akt1*^−/−^→*Ldlr*^−/−^ and WT→*Ldlr*^−/−^ mice (Fig. 1E). Importantly, recipient mice reconstituted with WT, *Akt1*^−/−^, or *Akt2*^−/−^ FLCs had similar numbers of apoptotic cells detected by TUNEL assay in atherosclerotic lesions (Fig. 1F). Thus, the deletion of Akt2, but not Akt1, in hematopoietic cells reduces early atherosclerosis in *Ldlr*^−/−^ recipient mice, in the absence of changes in serum lipid levels.

**TABLE 1.** Total serum cholesterol and triglyceride levels in *Ldlr*^−/−^ mice reconstituted with WT, *Akt1*^−/−^ , *Akt2*^−/−^ or FLCs and fed the Western diet for 8 or 16 weeks

Reconstituted Cell Types	Body Weight (g)	Cholesterol (mg/dl)	Triglycerides (mg/dl)
Females*^a^*			
WT (n = 9)	20.3 ± 0.9	846 ± 44	196 ± 11
Akt1^−/−^ (n = 10)	19.6 ± 0.8	777 ± 36	202 ± 19
Akt2^−/−^ (n = 10), *P*	20.3 ± 0.4, 0.75	815 ± 31, 0.44	207 ± 18, 0.90
Males*^a^*			
WT (n = 9)	27.9 ± 0.9	832 ± 52	242 ± 27
Akt1^−/−^ (n = 11), *P*	27.3 ± 0.7, 0.65	814 ± 55, 0.83	243 ± 22, 0.85
Males*^a^*			
WT (n = 10)	25.6 ± 0.3	990 ± 39	275 ± 14
Akt2^−/−^ (n = 10), *P*	25.2 ± 0.5, 0.47	930 ± 41, 0.15	226 ± 16, 0.11
Females*^b^*			
WT (n = 11)	22.7 ± 1.0	962 ± 52	217 ± 10
Akt1^−/−^ (n = 12)	23.3 ± 0.9	939 ± 36	203 ± 12
Akt2^−/−^ (n = 13), *P*	22.9 ± 1.0, 0.97	970 ± 42, 0.86	213 ± 23, 0.80

Values are in mg/dl (mean ± SEM). The number of recipient mice in each group is indicated by *n*. The differences are not statistically significant between the groups.

a Fed the Western diet for 8 weeks.

b Fed the Western diet for 16 weeks.

**Fig. 2.**
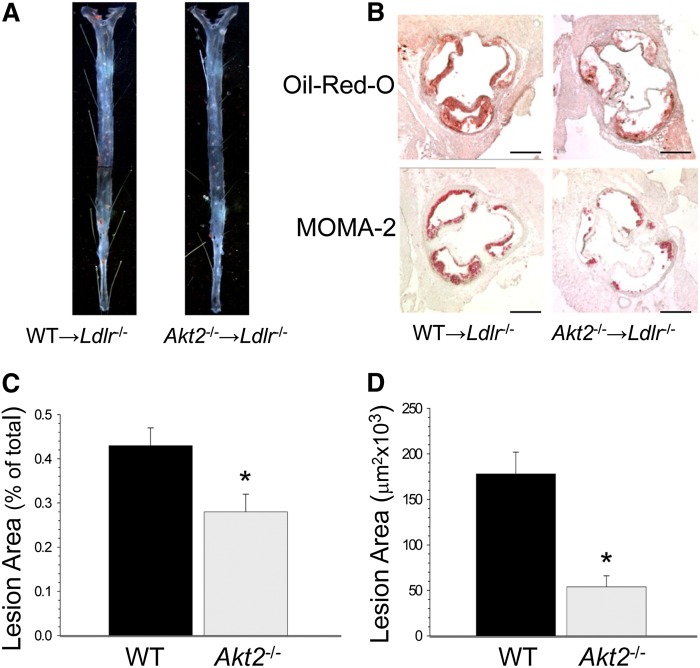
Hematopoietic *Akt2* deficiency suppresses early atherosclerosis in male *Ldlr*^−/−^mice. A, B: Atherosclerotic lesions in the distal (A) and proximal (B) aortas of mice reconstituted with WT and *Akt2*^−/−^ FLCs. Aortas were pinned out en face and stained with Sudan IV (A); serial sections of the aortic sinus were stained with Oil Red O or macrophage stain, MOMA-2 (B). Scale bars, 200 μm; a pin size, 10 μm. C, D: The extent of atherosclerotic lesions in the distal (C) and proximal (D) aortas of *Ldlr*^−/−^ mice reconstituted with WT (black) or *Akt2*^−/−^ (light gray) FLCs (* *P <* 0.05 by Mann-Whitney rank sum test).

To extend these findings, two additional experiments were performed. First, 19-week-old male *Ldlr*^−/−^ mice were transplanted with male WT (n = 9) or *Akt1*^−/−^ (n = 11) FLCs and, 4 weeks later, placed on the Western diet for 8 weeks. Recipient mice reconstituted with WT or *Akt1*^−/−^ FLCs did not differ significantly in body weight or serum levels of total cholesterol or triglycerides (Table 1). In addition, *Akt1*^−/−^→*Ldlr*^−/−^ and WT→*Ldlr*^−/−^ mice had nearly the same extent of atherosclerotic lesions (supplementary Fig. IA, B) with similarly sized lesion areas in the distal aortas pinned out en face (supplementary Fig. IC) and the aortic sinus (supplementary Fig. ID). In the second experiment, 15-week-old male *Ldlr*^−/−^ mice were reconstituted with male WT (n = 10) or *Akt2*^−/−^ (n = 10) FLCs and, 4 weeks later, fed with the Western diet for 8 weeks. The recipients had no differences in body weight or serum lipid levels (Table 1). However, *Akt2*^−/−^→*Ldlr*^−/−^ mice developed significantly smaller atherosclerotic lesions than WT→*Ldlr*^−/−^ mice, with a 34.9% reduction in lesion area in the distal aorta (0.28 *±* 0.04 vs. 0.43 ± 0.04%; **Fig. 2A, C**) and a 69.7% reduction in the proximal aorta (54.3 ± 12.1 vs. 178.4 ± 23.7 × 10^3^ μm^2^, Fig. 2B, D). *Akt2*^−/−^→*Ldlr*^−/−^ mice also had a 71.4% smaller area staining for macrophages in their atherosclerotic lesions than WT→*Ldlr*^−/−^ mice (48.3 ± 18.3 vs. 169.1 ± 6.4 × 10^3^ μm^2^). Taken together, these data demonstrate that hematopoietic deficiency of Akt2 reduces the extent of early atherosclerosis in *Ldlr*^−/−^ recipient mice of both genders.

In addition, because Akt2 deficiency significantly increases (3.3-fold) numbers of neutrophils in the blood (26) and neutrophils may impact atherogenesis (27), we analyzed the presence of these cells in atherosclerotic lesions of *Akt2*^−/−^→*Ldlr*^−/−^ and WT→*Ldlr*^−/−^ mice. Indeed, neutrophils were present in atherosclerotic lesions and adventitia of both types of recipients (supplementary Fig. IIA–F), but their numbers did not differ significantly between the groups (supplementary Fig. IIG).

### Akt2 deficiency in hematopoietic cells suppresses formation of advanced atherosclerotic lesions

**Fig. 3.**
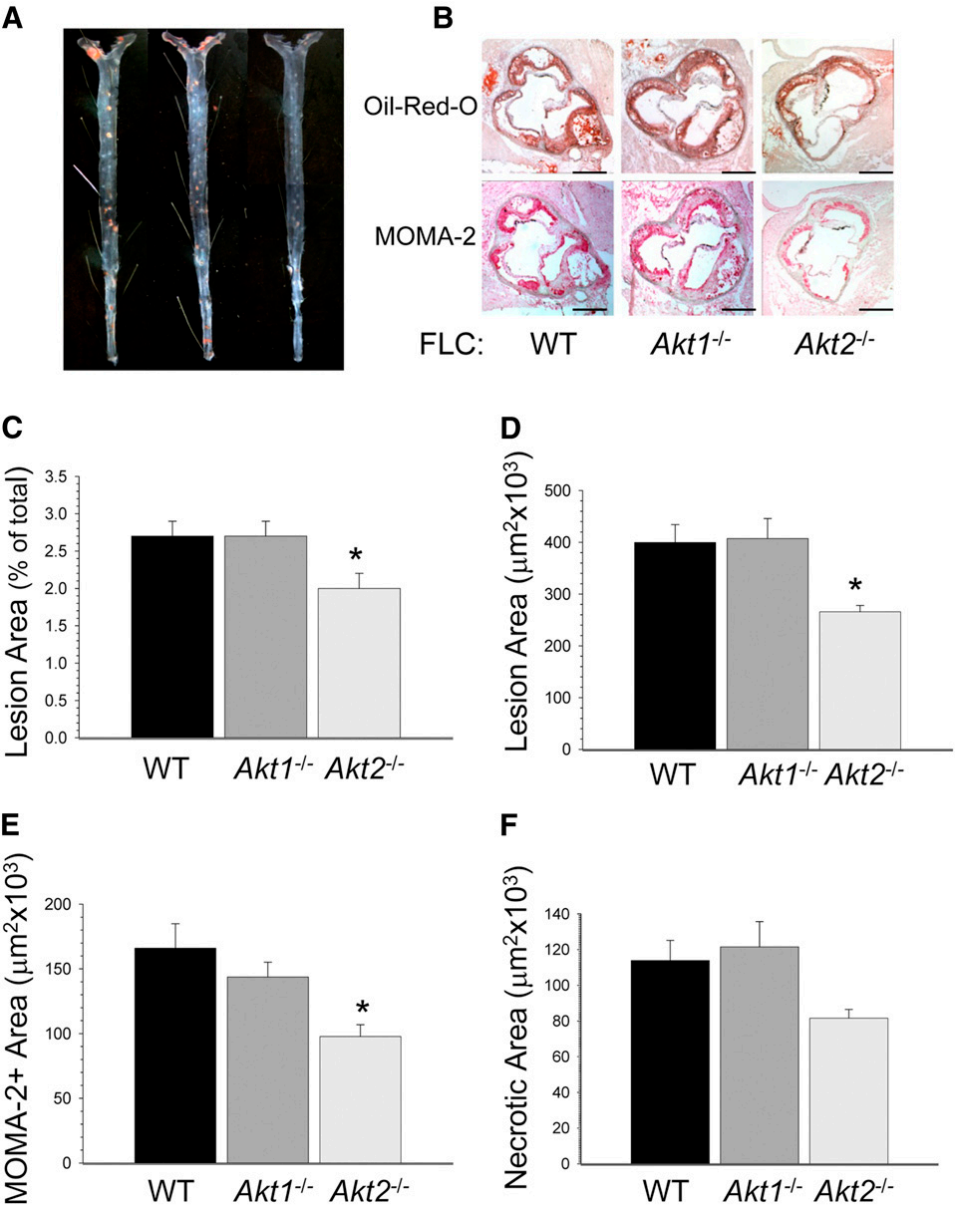
Loss of Akt2 in hematopoietic cells suppresses formation of advanced atherosclerotic lesions in *Ldlr*^−/−^ mice. A, B: Atherosclerotic lesions in the distal and proximal aortas of mice reconstituted with WT, *Akt1*^−/−^, or *Akt2*^−/−^ FLCs after 16 weeks on the Western diet. Mouse aortas pinned out en face (A) and aortic sinus sections stained with Oil Red O or MOMA-2 (B). Scale bars, 200 μm; a pin size, 10 μm. C–F: The extent of atherosclerotic lesions in the distal (C) and proximal (D) aortas, MOMA-2-positive area (E), and necrotic area (F) in atherosclerotic lesions of *Ldlr^−/−^* mice reconstituted with WT (black), *Akt1*^−/−^ (dark gray), or *Akt2*^−/−^ (light gray) FLCs (* *P <* 0.05 vs. control group by one-way ANOVA, Holm-Sidak method).

In the next set of experiments, female *Ldlr*^−/−^ mice reconstituted with WT (n = 11), *Akt1*^−/−^ (n = 12), or *Akt2*^−/−^ (n = 13) FLCs were fed the Western diet for 16 weeks to generate advanced atherosclerotic lesions. At euthanization, mice reconstituted with WT, *Akt1*^−/−^, or *Akt2*^−/−^ FLCs did not differ by body weight, blood glucose, or serum lipid levels (Table 1). Again, *Akt1*^−/−^→*Ldlr*^−/−^ and WT→*Ldlr*^−/−^ mice had similarly sized atherosclerotic lesions in the en face analysis of the distal aortas pinned out (**Fig. 3A, C**). In contrast, *Akt2*^−/−^→*Ldlr*^−/−^ mice developed significantly smaller (26%) atherosclerotic lesions in the distal aorta compared with WT→*Ldlr*^−/−^ and Akt1^−/−^→*Ldlr*^−/−^ mice. Similarly, *Akt2*^−/−^→*Ldlr*^−/−^ mice had smaller (by 33.5%) atherosclerotic lesions in the aortic sinus (Fig. 3B, D) compared with WT→*Ldlr*^−/−^ and Akt1^−/−^→*Ldlr*^−/−^ mice (265.6 ± 12.4 vs. 399.6 ± 34.5 and 407.2 ± 38.7 × 10^3^ μm^2^). These *Akt2*^−/−^→*Ldlr*^−/−^ mice also had smaller areas staining for macrophages in their atherosclerotic lesions than *Akt1*^−/−^→*Ldlr*^−/−^ and WT→*Ldlr*^−/−^ mice (Fig. 3E). Interestingly, the size of the necrotic area in atherosclerotic lesions of *Akt2*^−/−^→*Ldlr*^−/−^ mice was not statistically different from the necrotic area of WT→*Ldlr*^−/−^ and Akt1^−/−^→*Ldlr*^−/−^ mice (Fig. 3F). Furthermore, the percentage of total lesion area occupied by necrosis was similar between groups with ratios of 31, 29, and 30% in *Ldlr*^−/−^ mice reconstituted with WT, *Akt1*^−/−^, or *Akt2*^−/−^ FLCs, respectively. Because Akt2 is a negative regulator of the nuclear factor of activated T cells (28), we analyzed lymphocyte counts in aortic atherosclerotic lesions of recipient mice using an antibody to CD5 that detects all mature T lymphocytes and a subpopulation of activated B cells (supplementary Fig. IIIA–C). No differences in CD5-positive cells were found in atherosclerotic lesions or adventitia of mice transplanted with WT, *Akt1*^−/−^, or *Akt2*^−/−^ FLCs (supplementary Fig. IIID). Thus, Akt2 deficiency in hematopoietic cells has atheroprotective effects in both early and advanced atherosclerotic lesions of *Ldlr*^−/−^ mice.

### *Akt2*^−/−^→*Ldlr*^−/−^ mice have decreased levels of blood CCR2^+^/Ly6C^hi^ monocytes, and *Akt2*^−/−^ macrophages are polarized to M2 phenotype

**Fig. 4.**
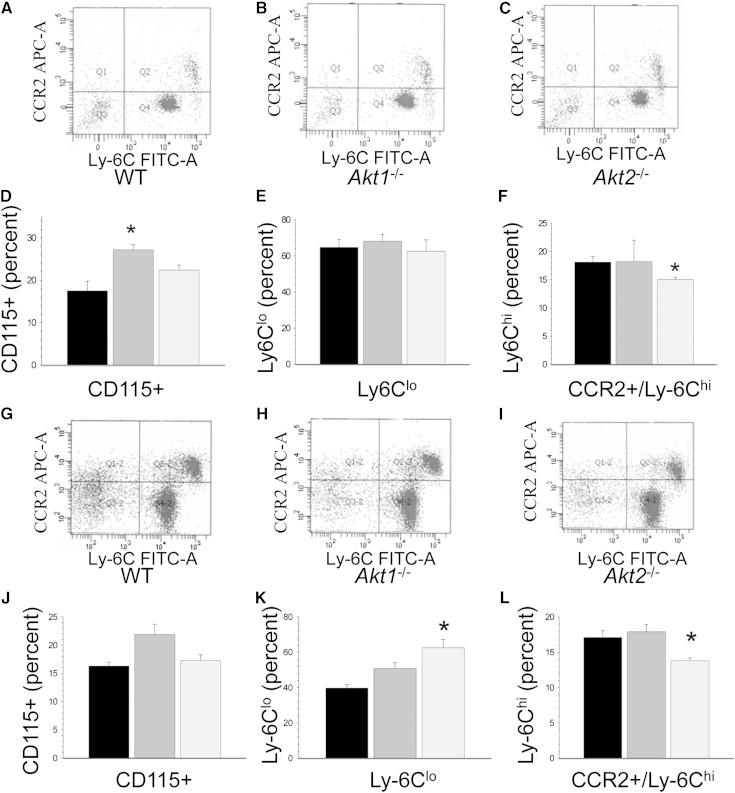
*Akt2*^−/−^→*Ldlr*^−/−^ mice have decreased levels of CCR2^+^/Ly6C^hi^ monocytes on a chow diet (A–F) and the Western diet (G–L). A–C, G–I: Flow cytometry analysis of CCR2 and Ly-6C expression in CD115-gated blood monocytes of *Ldlr*^−/−^ mice reconstituted with WT, *Akt1*^−/−^, and *Akt2*^−/−^ FLCs. D–F, J–L: Percent of CD115, Ly-6C^lo^, and CCR2^+^/Ly-6C^hi^ monocytes in mice transplanted with WT (black), *Akt1*^−/−^ (dark gray), and *Akt2*^−/−^ (light gray) FLCs (* *P* < 0.05 compared with WT cells by one-way ANOVA, Dunnett’s method).

Loss of Akt1 or Akt2 differentially contributes to macrophage polarization with *Akt1* deficiency giving rise to an M1 and Akt2 ablation resulting in an M2 phenotype (21). Therefore, we examined the expression of inflammatory markers in blood monocytes and macrophages of the recipient mice. On a chow diet, *Ldlr*^−/−^ recipient mice reconstituted with WT, *Akt1*^−/−^, and *Akt2*^−/−^ FLCs had similar levels of white blood cell counts (8.3 ± 0.5, 8.2 ± 0.7, and 10.2 ± 0.9 × 10^6^, respectively) and similar ratios of monocytes, B cells, and T cells in their blood (supplementary Fig. IV). Remarkably, *Akt1*^−/−^→*Ldlr*^−/−^ mice exhibited higher numbers of CD115-positive monocytes than WT→*Ldlr*^−/−^ and *Akt2*^−/−^→*Ldlr*^−/−^ mice (**Fig. 4D**). *Akt1*^−/−^→*Ldlr*^−/−^ mice also had higher levels of CD115^+^, iNOS^+^, and CCR2^+^/iNOS^+^ cells than WT→*Ldlr*^−/−^ and Akt2^−/−^→*Ldlr*^−/−^ mice (supplementary Fig. V). In contrast, *Akt2*^−/−^→*Ldlr*^−/−^ mice had significantly reduced levels of CCR2^+^/Ly-6C^hi^ monocytes (Fig. 4F), a known subset of inflammatory cells (29, 30). In addition, we examined the distribution of these markers on CD11b^+^ cells, which, in addition to monocytes, also stains granulocytes, natural killer cells, and subsets of T and B cells. After 4 weeks of Western diet, *Ldlr*^−/−^ mice (n = 4/group) reconstituted with WT, *Akt1*^−/−^, and *Akt2*^−/−^ FLCs had more Ly-6C^hi^ than Ly-6C^lo^ blood monocytes. Importantly, *Akt2*^−/−^→*Ldlr*^−/−^mice exhibited significantly fewer Ly-6C^hi^ monocytes, and *Akt1*^−/−^→*Ldlr*^−/−^ mice expressed more Ly-6C^hi^ monocytes than WT→*Ldlr*^−/−^ mice (supplementary Fig. VI). Similarly, *Akt1*^−/−^→*Ldlr*^−/−^ mice fed the Western diet exhibited a trend for an increase in the levels of CD115^+^ monocytes (Fig. 4J). Interestingly, *Akt2*^−/−^→*Ldlr*^−/−^ mice contained more Ly-6C^lo^ (Fig. 4K) and fewer CCR2^+^/Ly-6C^hi^ CD115^+^ monocytes than WT→*Ldlr*^−/−^ and *Akt1*^−/−^→*Ldlr*^−/−^ mice (Fig. 4L). These data indicate that *Akt1*^−/−^→*Ldlr*^−/−^ mice have higher and *Akt2*^−/−^→*Ldlr*^−/−^ mice have lower levels of inflammatory monocytes compared with control WT→*Ldlr*^−/−^ mice.

**Fig. 5.**
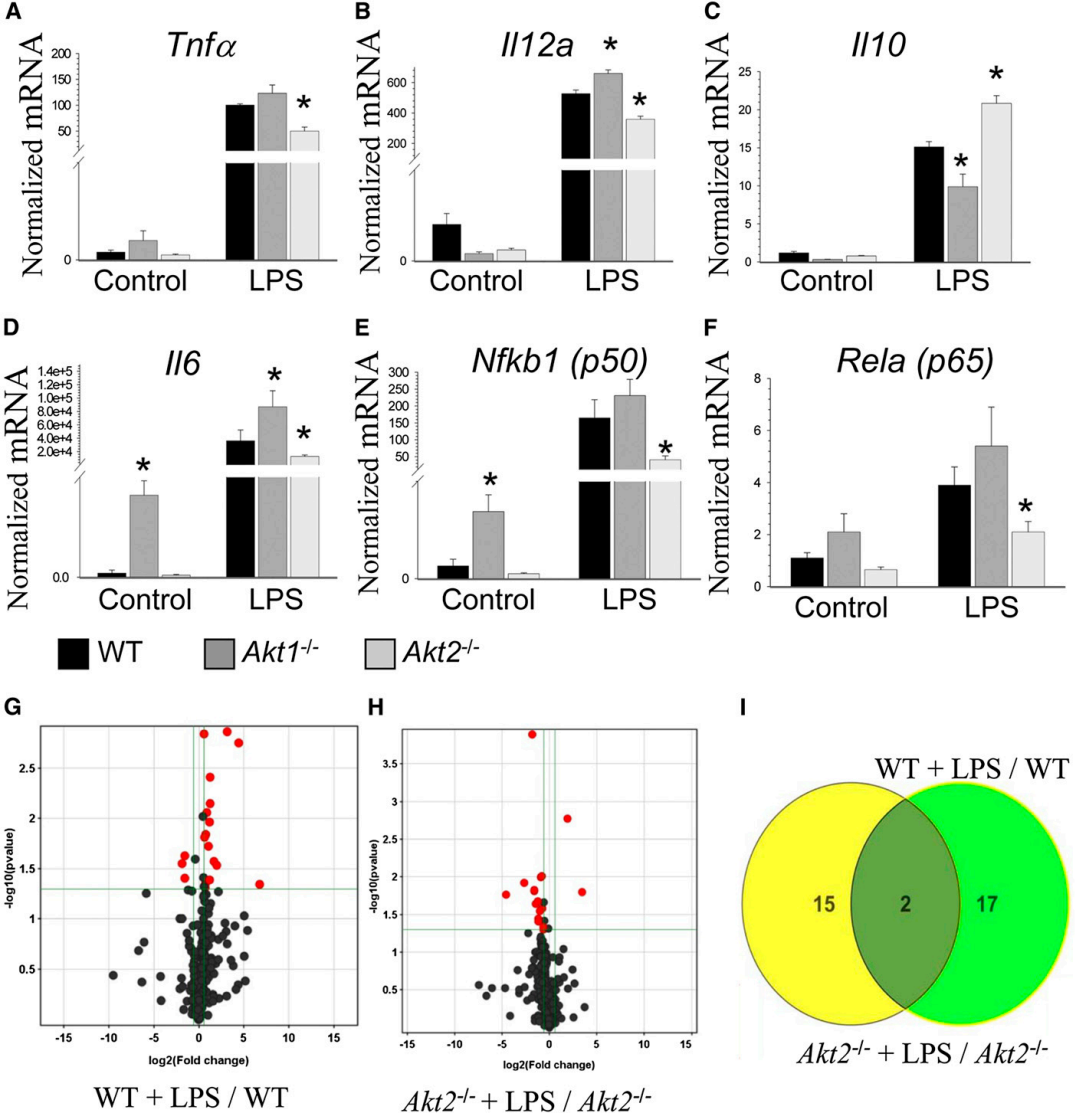
Distinct responses of *Akt1*^−/−^ and *Akt2*^−/−^ macrophages to LPS treatment compared with WT cells. A–F: Real-time PCR analysis of *Tnf*, *Il12a*,* Il10*,* Il6*,* Nfkb1 (p50)*, and* Rela(p60)* gene expression in WT (black), *Akt1*^−/−^ (dark gray), or *Akt2*^−/−^ (light gray) macrophages in response to LPS. Cells were incubated with media alone (control) or together with LPS (50 ng/ml) for 6 h. The graphs represent data (mean ± SEM) obtained from the same numbers (n = 3/group) of mice (* *P* < 0.05 compared with WT cells untreated or treated with LPS by one-way ANOVA). G, H: Volcano plots demonstrating (red dots) significantly (*P* < 0.05) altered (>1.5-fold absolute fold change) miRNA changes, reported as log2 (fold change) and –log10 (*P* value by unpaired *t*-test; n = 3). OpenArrays WT + LPS/ WT (G) and *Akt2^−/−^* + LPS/ *Akt2^−/−^* (H). I: Venn diagram highlighting 17 miRNAs that are altered by LPS and dependent on Akt2 in macrophages (green).

Akt signaling regulates diverse biological functions including cell survival, proliferation, and migration (2). In our studies, loss of Akt1 or Akt2 does not cause any apparent changes in Akt signaling of macrophages in response to insulin treatment (supplementary Fig. VII), supporting the notion of Akt isoform redundancy. Next, WT, *Akt1*^−/−^, and *Akt2*^−/−^ macrophages were treated with LPS, and the levels of proinflammatory genes were measured. Compared with WT cells, *Akt1*^−/−^ macrophages showed an increase in *Il12a* and *Il6* mRNA expression (**Fig. 5B, D**). On the other hand, Akt2^−/−^ macrophages exhibited significantly reduced expression of *Tnfα*, *Il12a*,* Il6*,* Nfkb1 (p50)*, and* Rela (p60)* (Fig. 5A–F). *Akt1*^−/−^ and *Akt2*^−/−^ macrophages significantly differed from WT cells by their *Il10* gene expression (Fig. 5C). Together these results indicate that *Akt1*^−/−^ and *Akt2*^−/−^ macrophages are skewed toward the M1 and M2 phenotypes, respectively.

Recent evidence suggests that miRNAs significantly contribute to macrophage polarization and immune responses (31, 32), including Akt-dependent processes (21). miRNAs are small noncoding RNAs that posttranscriptionally regulate gene expression through inhibition of translation and mRNA destabilization (33). To determine whether miRNAs contribute to Akt2-dependent macrophage polarization and response to activation, a systematic approach was used to identify macrophage miRNAs that are altered by LPS activation and dependent on *Akt2*. Using real-time PCR-based OpenArrays, we quantified the levels of 750 miRNAs and control small RNAs in peritoneal macrophages isolated from WT and *Akt2^−/−^* mice, treated with LPS or serum-free DMEM (control) for 6 h. In WT macrophages, we found that LPS significantly (*P* < 0.05) altered (>1.5-absolute fold change) 19 miRNAs, including 16 that were upregulated (Fig. 5G). Conversely, we found that LPS activation resulted in only 2 upregulated miRNAs out of 17 total significantly altered miRNAs in macrophages isolated from *Akt2^−/−^* mice (Fig. 5H). Due to the observed differences in response to LPS activation between WT and *Akt2^−/−^* mice macrophages, we filtered miRNAs that were significantly altered by LPS activation in macrophages from WT mice, but not from Akt2-deficient mice, and found 17 miRNAs that satisfied these criteria (Fig. 5I; supplementary Table I). These results strongly suggest that a majority of LPS-induced miRNA changes in macrophages are dependent on Akt2 and likely contribute to Akt2 control of macrophage phenotype.

### *Akt2*^−/−^ macrophages have restricted CCR2 expression and migration

**Fig. 6.**
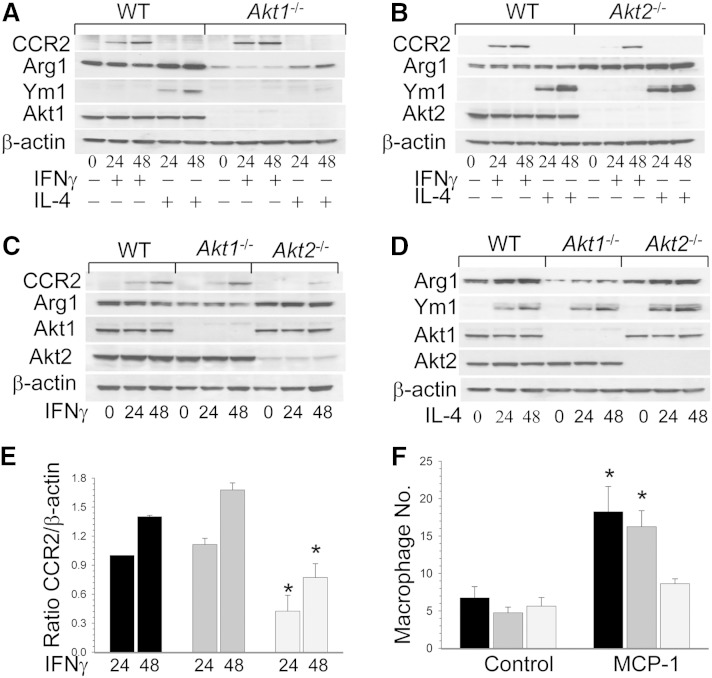
*Akt2*^−/−^ macrophages are resistant to M1 priming, and *Akt2*^−/−^ cells express less CCR2 and were slower in the migration assay than *Akt1*^−/−^ and WT cells. A–D: Expression of M1 and M2 markers in WT, *Akt1*^−/−^, and *Akt2*^−/−^ macrophages in response to IFNγ or IL-4. Peritoneal macrophages were untreated (0) or treated with the recombinant mouse IFNγ (50 ng/ml) or the recombinant mouse IL-4 (20 ng/ml) for 24 or 48 h. Macrophage proteins were extracted, resolved (40 μg/well), and analyzed by Western blot. E: The graphs exhibit the averages (mean ± SEM) of CCR2 expression in WT (black), *Akt1*^−/−^ (dark gray), or *Akt2*^−/−^ (light gray) macrophages after treatment with IFNγ (* *P* < 0.05 compared with control WT cells at the same time point by one-way ANOVA). F: Peritoneal macrophages were cultured in transwell inserts in triplicate for 2 h and, after addition of MCP-1 (100 ng/ml) into the lower chamber, incubated for 2 h at 37°C. The cells were stained with DAPI and analyzed under a fluorescent microscope (* *P <* 0.05 compared with control WT cells by one-way ANOVA, Tukey test).

To further investigate the impact of Akt isoform deficiency on macrophage polarization, WT, *Akt1*^−/−^, and *Akt2*^−/−^ peritoneal macrophages were primed to M1 or M2 phenotype by exposure to IFNγ or IL-4, respectively, and then studied for expression of a marker of classical activation, CCR2, and markers of alternative activation such as Arg1 and the chitinase-like molecule Ym1 (13). Compared with WT cells, *Akt1*^−/−^ macrophages expressed slightly more CCR2 after IFNγ treatment and showed reduced Arg1 and Ym1 expression after IL-4 treatment (**Fig. 6A**). In contrast, *Akt2*^−/−^ cells were skewed to the M2 phenotype, as they expressed more Arg1 and Ym1 after IL-4 treatment and less CCR2 in response to IFNγ than WT cells (Fig. 6B). Quantification of the Western blot data clearly demonstrates that *Akt1*^−/−^ macrophages express reduced levels of Arg1 and Ym1 compared with WT and *Akt2*^−/−^ cells (supplementary Fig. VIII). In contrast, *Akt2*^−/−^ macrophages had increased Arg1 and Ym1 levels compared with WT cells (supplementary Fig. VII). Remarkably, *Akt2*^−/−^ macrophages also had significantly less CCR2 expression than WT and *Akt1*^−/−^ cells when treated with IFNγ (Fig. 6E). This indicates that *Akt2*^−/−^ macrophages are resistant to priming toward the opposite M1 phenotype, which may significantly slow an inflammatory response.

Because CCR2 plays crucial roles in monocyte (34) and hematopoietic stem cell trafficking (35), we hypothesized that *Akt2*^−/−^ macrophages may have impaired mobility. To test this hypothesis, we compared WT, *Akt1*^−/−^, and *Akt2*^−/−^ macrophages in a monolayer injury assay. *Akt2*^−/−^ macrophages were significantly slower in recovering the monolayer than WT and *Akt1*^−/−^ cells. Furthermore, the Akt inhibitor IV (10 μM) and the PI3 kinase inhibitor wortmannin (50 nM) completely disrupted macrophage monolayer recovery (data not shown). Finally, a transwell migration assay demonstrated that treatment of WT, *Akt1*^−/−^, and *Akt2*^−/−^ peritoneal macrophages with MPC-1 significantly increased migration of WT and *Akt1*^−/−^, but not *Akt2*^−/−^ macrophages, which had lower (∼50%) levels of migration (Fig. 6F). Therefore, *Akt2*^−/−^ monocyte/macrophages exhibit reduced CCR2 expression and have impaired migration.

## DISCUSSION

Akt regulates a number of fundamental biological processes that play critical roles in the pathogenesis of atherosclerosis. Macrophages express three Akt isoforms, Akt1, Akt2, and Akt3, which play isoform-specific or redundant roles in Akt signaling. A recent report (11) described a nonredundant protective role for macrophage Akt3 in regulating lipoprotein uptake, with no effect on apoptosis. Interestingly, loss of Akt1 in *Apoe*^−/−^ mice results in accelerated atherosclerosis, due to a protective role of Akt1 in endothelial cells (9). However, the roles of macrophage expression of Akt1 and Akt2 in atherosclerosis remain unclear. Here we demonstrate that the loss of *Akt1* in hematopoietic cells of *Ldlr*^−/−^ mice had no effect on the extent of atherosclerotic lesions compared with control WT→*Ldlr*^−/−^ mice. In contrast, *Akt2*^−/−^→*Ldlr*^−/−^ mice of both genders had dramatically smaller early atherosclerotic lesions than either WT→*Ldlr*^−/−^ or *Akt1*^−/−^→*Ldlr*^−/−^ mice. These *Akt2*^−/−^→*Ldlr*^−/−^ mice had a smaller macrophage area but no changes in macrophage apoptosis in atherosclerotic lesions compared with lesions of WT→*Ldlr*^−/−^ or *Akt1*^−/−^→*Ldlr*^−/−^ mice. Similarly, *Akt2*^−/−^→*Ldlr*^−/−^ mice with advanced atherosclerotic lesions after 16 weeks on the Western diet showed reduced atherosclerotic lesions with a similar proportion of necrotic area in their lesions compared with WT→*Ldlr*^−/−^ or *Akt1*^−/−^→*Ldlr*^−/−^ mice. Importantly, *Akt2*^−/−^ macrophages were M2 skewed and had a restricted capability for M1 priming and CCR2 expression. Thus, loss of macrophage Akt2 expression has an isoform-specific impact on the development of atherosclerosis. This is the first in vivo demonstration that M2 polarization of macrophages achieved through genetic alteration of Akt2 has an impact on the development of atherosclerosis.

Akt plays critical roles in cell survival (2), but we did not see an impact of deficiency of either *Akt1* or *Akt2* in hematopoietic cells on apoptosis in the atherosclerotic lesions of *Ldlr*^−/−^ recipient mice after 8 or 16 weeks on the Western diet. Similarly, loss of Akt1 or Akt2 individually in embryonic fibroblasts (36) has no impact on apoptosis. In contrast, Akt1^−/−^/*Apoe*^−/−^ mice were reported to have increased TUNEL-positive macrophages in atherosclerotic lesions compared with control *Apoe*^−/−^ mice (9). The presence of apoE promotes cell survival factor (37), and we have recently shown that apoE deficiency in macrophages promotes apoptosis (38). Therefore, the *Apoe*^−/−^ background may have contributed to increased macrophage apoptosis in *Apoe*^−/−^/*Akt1*^−/−^ mice (9). In any case, loss of Akt1 or Akt2 in macrophages did not appear to impair cell survival in atherosclerotic lesions in the current study. Furthermore, the lack of a difference in the percentage of necrotic area in the atherosclerotic lesions is in keeping with the absence of an impact on apoptosis in the lesions.

Recently, Rensing and coauthors (12) reported that *Akt2*^−/−^/*Ldlr*^−/−^ DKO mice developed smaller carotid and aortic atherosclerotic lesions than Ldlr^−/−^controls, despite having significantly higher plasma total cholesterol, glucose, and insulin levels. These data are consistent with our results in terms of the impact on lesion size, but we have extended their findings by showing that loss of Akt2 expression in hematopoietic cells is sufficient to reduce the extent of atherosclerosis in *Ldlr^−/−^* mice. However, it is important to note that these *Akt2^−/−^/Ldlr^−/−^* DKO mice after 8 weeks of the Western diet developed more complex atherosclerotic lesions with only 15% macrophages and increased necrotic area compared with our *Akt2*^−/−^→*Ldlr*^−/−^ mice forming relatively small atherosclerotic lesions that consisted predominantly of macrophage-derived foam cells. Rensing et al. concluded that loss of Akt2 generates more complex morphology of atherosclerotic lesions in *Akt2*^−/−^/*Ldlr*^−/−^ mice as a result of suppression of vascular smooth muscle migration, proliferation, and collagen matrix production. Thus, the global loss of Akt2 expression in the *Akt2*^−/−^/*Ldlr*^−/−^ mice likely contributed to the more complex lesion formation compared with our study where Akt2 deficiency was confined to hematopoietic cells. Other contributing factors likely included the increased cholesterol in the diet (containing 0.25% cholesterol and 15% cocoa butter vs. our diet containing 0.15% cholesterol and 21% milk fat), as well as differences in blood glucose and insulin levels.

Human Akt1 and Akt2 isoforms share the same primary structure, with a high degree of homology in their nucleotide (77.5%) and amino acid (90.4%) sequences (39). The alignment of the primary amino acid sequences of human, rat, and mouse Akt1 and Akt2 shows a high level (98%) of homology, with substantial differences between the isoforms only in the last 130 amino acids (40). Recently, Arranz et al. (21) demonstrated that Akt1 and Akt2 play opposing roles in macrophage polarization, with Akt1 ablation generating the M1 phenotype and Akt2 deficiency skewing cells toward the M2 phenotype. Interestingly, *Akt1*^−/−^ mice are more sensitive and *Akt2*^−/−^ mice are more resistant than WT mice to LPS-induced endotoxin shock, and adoptive transfer of WT macrophages restores the normal inflammatory reaction (21). Our results strongly support the contention that *Akt1*^−/−^ blood monocytes and macrophages are skewed to the M1 phenotype, and *Akt2*^−/−^ monocyte/macrophages display the M2 phenotype. We consistently found that *Akt2*^−/−^ monocytes and macrophages expressed significantly lower levels of inflammatory genes. In related work, SH2-containing inositol 5-phosphatase (SHIP), which is a potent negative regulator of the PI3K pathway, has been reported to repress the generation of M2 macrophages (19, 41). Thus, SHIP^−/−^ peritoneal macrophages display the M2 phenotype with low IL-12 and high IL-10 expression in response to LPS (19, 41). In addition, *Akt2*^−/−^ macrophages exhibited a suppressed ability for M1 polarization and CCR2 induction when treated with IFNγ. Therefore, *Akt2*^−/−^ macrophages exhibited suppressed migration in response to MCP-1. Similarly, *Akt2*^−/−^ neutrophils have been reported to exhibit decreased migration compared with WT cells (26), and Akt2 inhibition reduces aggregation of neutrophils and platelets isolated from patients with sickle cell disease (42). It is possible that neutrophils may have contributed to the impact of Akt2 deficiency on atherosclerosis, but we did not see differences in the numbers of neutrophils in early lesions (supplementary Fig. II). Supporting an important role for Akt2 in cell migration, Akt2 overexpression upregulates β1 integrin, which increases invasion and metastasis of human breast and ovarian cancer cells (43, 44). Several studies have highlighted the distinct or opposing functions of Akt1 and Akt2 in Rac/Pak signaling and cell migration (45, 46), in the initiation of invasion and metastasis of tumor cancer cells (47, 48), and in cell proliferation (49). Together, these data support the critical importance of Akt1 and Akt2 isoforms in macrophage polarization, which may control a number of cell functions including immune responses, migration, and recruitment (17).

There is mounting evidence that changes in monocyte and macrophage phenotype significantly influence the initiation and progression of atherosclerosis (50). A recent concept (16, 22) proposes that an imbalance in the ratio of classically and alternatively activated macrophages plays a major role in atherogenesis. For example, Sharma and coworkers (51) reported that macrophages from mice deficient for Krüppel-like factor 4, which promotes M2 polarization, are skewed to the M1 type and develop increased atherosclerosis. In contrast, the administration of IL-13 to *Ldlr*^−/−^ mice polarized macrophages to the M2 state and had atheroprotective effects (52). Similarly, injections of thioredoxin-1, which is an oxidative stress-limiting protein, shifted macrophages to the M2 phenotype, and this suppressed aortic lesion formation in *Apoe*^−/−^ mice (53). Here we present a unique insight into the mechanisms and physiological relevance of macrophage polarization in atherosclerosis: Akt1 and Akt2 isoforms are highly homologous and functionally redundant in Akt signaling, but genetic deletion of these isoforms in macrophages results in the development of isoform-specific opposing phenotypes with differential effects in both early and advanced atherosclerosis. In our studies, both male and female *Ldlr*^−/−^ mice reconstituted with Akt1^−/−^ hematopoietic cells had similarly sized atherosclerotic lesions compared with control WT→*Ldlr*^−/−^ mice. The lack of impact of macrophage Akt1 on the extent of atherosclerosis is consistent with the observation that *Akt1*^−/−^*Apoe*^−/−^ bone marrow was not sufficient to worsen atherogenesis in *Apoe*^−/−^ recipient mice (9). We have extended these findings to *Ldlr*^−/−^ mice, a model of familial hypercholesterolemia (54).

It is clear that several functional patterns of M1 macrophages can be activated in response to inflammatory stimuli (15), and each phenotype may have a different impact on atherogenesis. In contrast, mice reconstituted with *Akt2*^−/−^ marrow had dramatically reduced atherosclerosis compared with WT→*Ldlr*^−/−^ controls and Akt1^−/−^→*Ldlr*^−/−^ mice. Our results are consistent with the previous reports indicating that Akt2 is required for macrophage chemotaxis (55) and that Akt2 deficiency impairs macrophage motility in a mouse myocardial injury model (56). Akt2 deficiency associated with the M2 phenotype can also contribute to atherosclerosis regression (57). Although a number of other factors may also regulate the mobility of immune cells, the CCR2/MCP-1 axis appears to be crucial for monocyte mobilization (34) and hematopoietic stem cell trafficking to sites of inflammation (35). Remarkably, *Akt2*^−/−^→*Ldlr*^−/−^ mice had decreased numbers of proinflammatory monocytes expressing CCR2^+^/Ly-C6^hi^, the subset of monocytes that efficiently accumulates in atherosclerotic lesions (29, 30). Recently, nanoparticle-facilitated siRNA silencing of CCR2 significantly reduced this inflammatory subset of monocytes, inhibited their accumulation in sites of inflammation, and suppressed the development of atherosclerotic lesions (58) and myocardial healing (59) in *Apoe*^−/−^ mice. Moreover, we found that LPS activation results in profound differential miRNA changes in macrophages from WT and Akt2^−/−^ mice. Specifically, we found that many LPS-altered miRNAs are dependent on Akt2, as they were not significantly affected by LPS in macrophages from *Akt2^−/−^* mice. Interestingly, specific Akt2-repressed LPS-induced miRNAs, miR-126-5p and miR-362-5p, are predicted to target and suppress Ccr2 in mice, and this could contribute to the observed loss in CCR2 expression in *Akt2*-deficient macrophages. Likewise, mmu-miR-125a-5p was found to be significantly upregulated in macrophages from WT mice upon LPS stimulation, but not in *Akt2*-deficient macrophages. After LPS induction, *Tnf* levels were found to be significantly reduced in macrophages from *Akt2^−/−^* mice compared with WT mice. Strikingly, miR-125a-5p is predicted to target and repress *Tnf* expression (mRNA levels) in both humans and mice. These results support the concept that Akt2 suppresses specific miRNAs that may target critical macrophage polarization and phenotypic regulators. Collectively, miRNA results presented here demonstrate that macrophage miRNA regulation is highly dependent on Akt2 regulation, namely in response to LPS activation. Nevertheless, future studies will be required to fully define Akt2-regulated miRNA contributions to macrophage phenotypes through specific mRNA targets. Taken together, these results suggest that modulation of monocyte/macrophage phenotype and CCR2 expression may be a promising strategy to treat atherosclerotic vascular disease.

In conclusion, we have demonstrated that hematopoietic deficiency of Akt2 reduces formation of early and advanced atherosclerosis in *Ldlr*^−/−^ recipient mice. Akt2 deficiency promotes M2 polarization and suppresses M1 priming, which reduces CCR2 induction, resulting in decreased macrophage migration to MCP-1. Our results demonstrate a critical isoform-specific role of Akt2 in macrophage polarization and atherogenesis in vivo, thus suggesting new therapeutic targets for the prevention and treatment of atherosclerosis.

## Supplementary Material

Supplemental Data

## References

[1] Moore K. J. Sheedy F. J. Fisher E. A. 2013 Macrophages in atherosclerosis: a dynamic balance. Nat. Rev. Immunol. 13: 709–721. 23995626 10.1038/nri3520 PMC4357520

[2] Manning B. D. Cantley L. C. 2007 AKT/PKB signaling: navigating downstream. Cell. 129: 1261–1274. 17604717 10.1016/j.cell.2007.06.009 PMC2756685

[3] Liu H. Perlman H. Pagliari L. J. Pope R. M. 2001 Constitutively activated Akt-1 is vital for the survival of human monocyte-differentiated macrophages. Role of Mcl-1, independent of nuclear factor (NF)-kappaB, Bad, or caspase activation. J. Exp. Med. 194: 113–126. 11457886 10.1084/jem.194.2.113 PMC2193455

[4] Chen W. S. Xu P-Z. Gottlob K. Chen M-L. Sokol K. Shiyanova T. Roninson I. Weng W. Suzuki R. Tobe K. 2001 Growth retardation and increased apoptosis in mice with homozygous disruption of the Akt1 gene. Genes Dev. 15: 2203–2208. 11544177 10.1101/gad.913901 PMC312770

[5] Cho H. Thorvaldsen J. L. Chu Q. Feng F. Birnbaum M. J. 2001 Akt1/PKBalpha is required for normal growth but dispensable for maintenance of glucose homeostasis in mice. J. Biol. Chem. 276: 38349–38352. 11533044 10.1074/jbc.C100462200

[6] Cho H. Mu J. Kim J. K. Thorvaldsen J. L. Chu Q. Crenshaw E. B. III Kaestner K. H. Bartolomei M. S. Shulman G. I. Birnbaum M. J. 2001 Insulin resistance and a diabetes mellitus-like syndrome in mice lacking the protein kinase Akt2 (PKB beta). Science. 292: 1728–1731. 11387480 10.1126/science.292.5522.1728

[7] Easton R. M. Cho H. Roovers K. Shineman D. W. Mizrahi M. Forman M. S. Lee V. M-Y. Szabolcs M. de Jong R. Oltersdorf T. 2005 Role for Akt3/protein kinase Bγ in attainment of normal brain size. Mol. Cell. Biol. 25: 1869–1878. 15713641 10.1128/MCB.25.5.1869-1878.2005 PMC549378

[8] Fujio Y. Walsh K. 1999 Akt mediates cytoprotection of endothelial cells by vascular endothelial growth factor in an anchorage-dependent manner. J. Biol. Chem. 274: 16349–16354. 10347193 10.1074/jbc.274.23.16349 PMC3624707

[9] Fernández-Hernando C. Ackah E. Yu J. Suárez Y. Murata T. Iwakiri Y. Prendergast J. Miao R. Q. Birnbaum M. J. Sessa W. C. 2007 Loss of Akt1 leads to severe atherosclerosis and occlusive coronary artery disease. Cell Metab. 6: 446–457. 18054314 10.1016/j.cmet.2007.10.007 PMC3621848

[10] Fernández-Hernando C. József L. Jenkins D. Di Lorenzo A. Sessa W. C. 2009 Absence of Akt1 reduces vascular smooth muscle cell migration and survival and induces features of plaque vulnerability and cardiac dysfunction during atherosclerosis. Arterioscler. Thromb. Vasc. Biol. 29: 2033–2040. 19762778 10.1161/ATVBAHA.109.196394 PMC2796372

[11] Ding L. Biswas S. Morton R. E. Smith J. D. Hay N. Byzova T. V. Febbraio M. Podrez E. A. 2012 Akt3 deficiency in macrophages promotes foam cell formation and atherosclerosis in mice. Cell Metab. 15: 861–872. 22632897 10.1016/j.cmet.2012.04.020 PMC3372639

[12] Rensing K. L. de Jager S. C. A. Stroes E. S. Vos M. Twickler M. T. B. Dallinga-Thie G. M. de Vries C. J. M. Kuiper J. Bot I. von der Thüsen J. H. 2014 Akt2/LDLr double knockout mice display impaired glucose tolerance and develop more complex atherosclerotic plaques than LDLr knockout mice. Cardiovasc. Res. 101: 277–287. 24220638 10.1093/cvr/cvt252

[13] Gordon S. Martinez F. O. 2010 Alternative activation of macrophages: mechanism and functions. Immunity. 32: 593–604. 20510870 10.1016/j.immuni.2010.05.007

[14] Mantovani A. Sica A. Sozzani S. Allavena P. Vecchi A. Locati M. 2004 The chemokine system in diverse forms of macrophage activation and polarization. Trends Immunol. 25: 677–686. 15530839 10.1016/j.it.2004.09.015

[15] Stout R. D. Jiang C. Matta B. Tietzel I. Watkins S. K. Suttles J. 2005 Macrophages sequentially change their functional phenotype in response to changes in microenvironmental influences. J. Immunol. 175: 342–349. 15972667 10.4049/jimmunol.175.1.342

[16] Mantovani A. Garlanda C. Locati M. 2009 Macrophage diversity and polarization in atherosclerosis. Arterioscler. Thromb. Vasc. Biol. 29: 1419–1423. 19696407 10.1161/ATVBAHA.108.180497

[17] Shalhoub J. Falck-Hansen M. Davies A. Monaco C. 2011 Innate immunity and monocyte-macrophage activation in atherosclerosis. J. Inflamm. (Lond.). 8: 9. 21526997 10.1186/1476-9255-8-9 PMC3094203

[18] Feig J. E. Parathath S. Rong J. X. Mick S. L. Vengrenyuk Y. Grauer L. Young S. G. Fisher E. A. 2011 Reversal of hyperlipidemia with a genetic switch favorably affects the content and inflammatory state of macrophages in atherosclerotic plaques. Circulation. 123: 989–998. 21339485 10.1161/CIRCULATIONAHA.110.984146 PMC3131163

[19] Rauh M. J. 2005 SHIP represses the generation of alternatively activated macrophages. Immunity. 23: 361–374. 16226502 10.1016/j.immuni.2005.09.003

[20] Vanhaesebroeck B. Jones G. E. Allen W. E. Zicha D. Hooshmand-Rad R. Sawyer C. Wells C. Waterfield M. D. Ridley A. J. 1999 Distinct PI(3)Ks mediate mitogenic signalling and cell migration in macrophages. Nat. Cell Biol. 1: 69–71. 10559867 10.1038/9045

[21] Arranz A. Doxaki C. Vergadi E. Martinez de la Torre Y. Vaporidi K. Lagoudaki E. D. Ieronymaki E. Androulidaki A. Venihaki M. Margioris A. N. 2012 Akt1 and Akt2 protein kinases differentially contribute to macrophage polarization. Proc. Natl. Acad. Sci. USA. 109: 9517–9522. 22647600 10.1073/pnas.1119038109 PMC3386059

[22] Tabas I. 2010 Macrophage death and defective inflammation resolution in atherosclerosis. Nat. Rev. Immunol. 10: 36–46. 19960040 10.1038/nri2675 PMC2854623

[23] Leavens K. F. Easton R. M. Shulman G. I. Previs S. F. Birnbaum M. J. 2009 Akt2 is required for hepatic lipid accumulation in models of insulin resistance. Cell Metab. 10: 405–418. 19883618 10.1016/j.cmet.2009.10.004 PMC2796129

[24] Babaev V. R. Fazio S. Gleaves L. A. Carter K. J. Semenkovich C. F. Linton M. F. 1999 Macrophage lipoprotein lipase promotes foam cell formation and atherosclerosis in vivo. J. Clin. Invest. 103: 1697–1705. 10377176 10.1172/JCI6117 PMC408384

[25] Babaev V. R. Chew J. D. Ding L. Davis S. Breyer M. D. Breyer R. M. Oates J. A. Fazio S. Linton M. F. 2008 Macrophage EP4 deficiency increases apoptosis and suppresses early atherosclerosis. Cell Metab. 8: 492–501. 19041765 10.1016/j.cmet.2008.09.005 PMC2614698

[26] Chen J. Tang H. Hay N. Xu J. Ye R. D. 2010 Akt isoforms differentially regulate neutrophil functions. Blood. 115: 4237–4246. 20332370 10.1182/blood-2009-11-255323 PMC2879106

[27] Soehnlein O. 2012 Multiple roles for neutrophils in atherosclerosis. Circ. Res. 110: 875–888. 22427325 10.1161/CIRCRESAHA.111.257535

[28] Martin V. A. Wang W-H. Lipchik A. M. Parker L. L. He Y. Zhang S. Zhang Z-Y. Geahlen R. L. 2012 Akt2 inhibits the activation of NFAT in lymphocytes by modulating calcium release from intracellular stores. Cell. Signal. 24: 1064–1073. 22261254 10.1016/j.cellsig.2012.01.001 PMC3289042

[29] Swirski F. K. Libby P. Aikawa E. Alcaide P. Luscinskas F. W. Weissleder R. Pittet M. J. 2007 Ly-6Chi monocytes dominate hypercholesterolemia-associated monocytosis and give rise to macrophages in atheromata. J. Clin. Invest. 117: 195–205. 17200719 10.1172/JCI29950 PMC1716211

[30] Tacke F. Alvarez D. Kaplan T. J. Jakubzick C. Spanbroek R. Llodra J. Garin A. Liu J. Mack M. van Rooijen N. 2007 Monocyte subsets differentially employ CCR2, CCR5, and CX3CR1 to accumulate within atherosclerotic plaques. J. Clin. Invest. 117: 185–194. 17200718 10.1172/JCI28549 PMC1716202

[31] Graff J. W. Dickson A. M. Clay G. McCaffrey A. P. Wilson M. E. 2012 Identifying functional microRNAs in macrophages with polarized phenotypes. J. Biol. Chem. 287: 21816–21825. 22549785 10.1074/jbc.M111.327031 PMC3381144

[32] Liu G. Abraham E. 2013 MicroRNAs in immune response and macrophage polarization. Arterioscler. Thromb. Vasc. Biol. 33: 170–177. 23325473 10.1161/ATVBAHA.112.300068 PMC3549532

[33] Baek D. Villén J. Shin C. Camargo F. D. Gygi S. P. Bartel D. P. 2008 The impact of microRNAs on protein output. Nature. 455: 64–71. 18668037 10.1038/nature07242 PMC2745094

[34] Tsou C-L. Peters W. Si Y. Slaymaker S. Aslanian A. M. Weisberg S. P. Mack M. Charo I. F. 2007 Critical roles for CCR2 and MCP-3 in monocyte mobilization from bone marrow and recruitment to inflammatory sites. J. Clin. Invest. 117: 902–909. 17364026 10.1172/JCI29919 PMC1810572

[35] Si Y. Tsou C-L. Croft K. Charo I. F. 2010 CCR2 mediates hematopoietic stem and progenitor cell trafficking to sites of inflammation in mice. J. Clin. Invest. 120: 1192–1203. 20234092 10.1172/JCI40310 PMC2846049

[36] Liu X. Shi Y. Birnbaum M. J. Ye K. De Jong R. Oltersdorf T. Giranda V. L. Luo Y. 2006 Quantitative analysis of anti-apoptotic function of Akt in Akt1 and Akt2 double knock-out mouse embryonic fibroblast cells under normal and stressed conditions. J. Biol. Chem. 281: 31380–31388. 16923802 10.1074/jbc.M606603200

[37] Beffert U. Nematollah Farsian F. Masiulis I. Hammer R. E. Yoon S. O. Giehl K. M. Herz J. 2006 ApoE receptor 2 controls neuronal survival in the adult brain. Curr. Biol. 16: 2446–2452. 17174920 10.1016/j.cub.2006.10.029

[38] Yancey P. G. Ding Y. Fan D. Blakemore J. L. Zhang Y. Ding L. Zhang J. Linton M. F. Fazio S. 2011 Low-density lipoprotein receptor–related protein 1 prevents early atherosclerosis by limiting lesional apoptosis and inflammatory Ly-6Chigh monocytosis: evidence that the effects are not apolipoprotein E dependent. Circulation. 124: 454–464. 21730304 10.1161/CIRCULATIONAHA.111.032268 PMC3144781

[39] Cheng J. Q. Godwin A. K. Bellacosa A. Taguchi T. Franke T. F. Hamilton T. C. Tsichlis P. N. Testa J. R. 1992 AKT2, a putative oncogene encoding a member of a subfamily of protein-serine/threonine kinases, is amplified in human ovarian carcinomas. Proc. Natl. Acad. Sci. USA. 89: 9267–9271. 1409633 10.1073/pnas.89.19.9267 PMC50107

[40] Heron-Milhavet L. Khouya N. Fernandez A. Lamb N. J. 2011 Akt1 and Akt2: differentiating the aktion. Histol. Histopathol. 26: 651–662. 21432781 10.14670/HH-26.651

[41] Kuroda E. Ho V. Ruschmann J. Antignano F. Hamilton M. Rauh M. J. Antov A. Flavell R. A. Sly L. M. Krystal G. 2009 SHIP represses the generation of IL-3-induced M2 macrophages by inhibiting IL-4 production from basophils. J. Immunol. 183: 3652–3660. 19710468 10.4049/jimmunol.0900864

[42] Li J. Kim K. Hahm E. Molokie R. Hay N. Gordeuk V. R. Du X. Cho J. 2014 Neutrophil AKT2 regulates heterotypic cell-cell interactions during vascular inflammation. J. Clin. Invest. 124: 1483–1496. 24642468 10.1172/JCI72305 PMC3973084

[43] Arboleda M. J. Lyons J. F. Kabbinavar F. F. Bray M. R. Snow B. E. Ayala R. Danino M. Karlan B. Y. Slamon D. J. 2003 Overexpression of AKT2/protein kinase Bβ leads to up-regulation of β1 integrins, increased invasion, and metastasis of human breast and ovarian cancer cells. Cancer Res. 63: 196–206. 12517798

[44] Cheng G. Z. Chan J. Wang Q. Zhang W. Sun C. D. Wang L-H. 2007 Twist transcriptionally up-regulates AKT2 in breast cancer cells leading to increased migration, invasion, and resistance to paclitaxel. Cancer Res. 67: 1979–1987. 17332325 10.1158/0008-5472.CAN-06-1479

[45] Irie H. Y. Pearline R. V. Grueneberg D. Hsia M. Ravichandran P. Kothari N. Natesan S. Brugge J. S. 2005 Distinct roles of Akt1 and Akt2 in regulating cell migration and epithelial-mesenchymal transition. J. Cell Biol. 171: 1023–1034. 16365168 10.1083/jcb.200505087 PMC2171329

[46] Zhou G-L. Tucker D. F. Bae S. S. Bhatheja K. Birnbaum M. J. Field J. 2006 Opposing roles for Akt1 and Akt2 in Rac/Pak signaling and cell migration. J. Biol. Chem. 281: 36443–36453. 17012749 10.1074/jbc.M600788200

[47] Dillon R. L. Marcotte R. Hennessy B. T. Woodgett J. R. Mills G. B. Muller W. J. 2009 Akt1 and Akt2 play distinct roles in the initiation and metastatic phases of mammary tumor progression. Cancer Res. 69: 5057–5064. 19491266 10.1158/0008-5472.CAN-08-4287 PMC4151524

[48] Chin Y. R. Toker A. 2011 Akt isoform-specific signaling in breast cancer: uncovering an anti-migratory role for palladin. Cell Adh. Migr. 5: 211–214. 21519185 10.4161/cam.5.3.15790 PMC3210203

[49] Héron-Milhavet L. Franckhauser C. Rana V. Berthenet C. Fisher D. Hemmings B. A. Fernandez A. Lamb N. J. C. 2006 Only Akt1 is required for proliferation, while Akt2 promotes cell cycle exit through p21 binding. Mol. Cell. Biol. 26: 8267–8280. 16982699 10.1128/MCB.00201-06 PMC1636765

[50] Ley K. Miller Y. I. Hedrick C. C. 2011 Monocyte and macrophage dynamics during atherogenesis. Arterioscler. Thromb. Vasc. Biol. 31: 1506–1516. 21677293 10.1161/ATVBAHA.110.221127 PMC3133596

[51] Sharma N. Lu Y. Zhou G. Liao X. Kapil P. Anand P. Mahabeleshwar G. H. Stamler J. S. Jain M. K. 2012 Myeloid Krüppel-like factor 4 deficiency augments atherogenesis in ApoE−/− mice—brief report. Arterioscler. Thromb. Vasc. Biol. 32: 2836–2838. 23065827 10.1161/ATVBAHA.112.300471 PMC3574634

[52] Cardilo-Reis L. Gruber S. Schreier S. M. Drechsler M. Papac-Milicevic N. Weber C. Wagner O. Stangl H. Soehnlein O. Binder C. J. 2012 Interleukin-13 protects from atherosclerosis and modulates plaque composition by skewing the macrophage phenotype. EMBO Mol. Med. 4: 1072–1086. 23027612 10.1002/emmm.201201374 PMC3491837

[53] El Hadri K. Mahmood D. F. D. Couchie D. Jguirim-Souissi I. Genze F. Diderot V. Syrovets T. Lunov O. Simmet T. Rouis M. 2012 Thioredoxin-1 promotes anti-inflammatory macrophages of the M2 phenotype and antagonizes atherosclerosis. Arterioscler. Thromb. Vasc. Biol. 32: 1445–1452. 22516068 10.1161/ATVBAHA.112.249334

[54] Ishibashi S. Brown M. S. Goldstein J. L. Gerard R. D. Hammer R. E. Herz J. 1993 Hypercholesterolemia in low density lipoprotein receptor knockout mice and its reversal by adenovirus-mediated gene delivery. J. Clin. Invest. 92: 883–893. 8349823 10.1172/JCI116663 PMC294927

[55] Zhang B. Ma Y. Guo H. Sun B. Niu R. Ying G. Zhang N. 2009 Akt2 is required for macrophage chemotaxis. Eur. J. Immunol. 39: 894–901. 19197940 10.1002/eji.200838809

[56] Li X. Mikhalkova D. Gao E. Zhang J. Myers V. Zincarelli C. Lei Y. Song J. Koch W. J. Peppel K. 2011 Myocardial injury after ischemia-reperfusion in mice deficient in Akt2 is associated with increased cardiac macrophage density. Am. J. Physiol. Heart Circ. Physiol. 301: H1932–H1940. 21890689 10.1152/ajpheart.00755.2010 PMC3213957

[57] Feig J. E. Vengrenyuk Y. Reiser V. Wu C. Statnikov A. Aliferis C. F. Garabedian M. J. Fisher E. A. Puig O. 2012 Regression of atherosclerosis is characterized by broad changes in the plaque macrophage transcriptome. PLoS ONE. 7: e39790. 22761902 10.1371/journal.pone.0039790 PMC3384622

[58] Leuschner F. Dutta P. Gorbatov R. Novobrantseva T. I. Donahoe J. S. Courties G. Lee K. M. Kim J. I. Markmann J. F. Marinelli B. 2011 Therapeutic siRNA silencing in inflammatory monocytes in mice. Nat. Biotechnol. 29: 1005–1010. 21983520 10.1038/nbt.1989 PMC3212614

[59] Majmudar M. D. Keliher E. J. Heidt T. Leuschner F. Truelove J. Sena B. F. Gorbatov R. Iwamoto Y. Dutta P. Wojtkiewicz G. 2013 Monocyte-directed RNAi targeting CCR2 improves infarct healing in atherosclerosis-prone mice. Circulation. 127: 2038–2046. 23616627 10.1161/CIRCULATIONAHA.112.000116 PMC3661714

