# Supplementary material for: Macrophage deficiency of Akt2 reduces atherosclerosis in Ldlr null mice
Source: J Lipid Res. 2014 Nov;55(11):2296–308. doi: 10.1194/jlr.M050633 (PMC4617132; doi:10.1194/jlr.M050633)
Supplement: Supplemental Data [file supp_55_11_2296__index.html]

Macrophage deficiency of Akt2 reduces atherosclerosis in LDLR null mice — Macrophage deficiency of Akt2 reduces atherosclerosis in Ldlr null mice — Supplemental Data 

# Macrophage deficiency of Akt2 reduces atherosclerosis in *Ldlr* null mice

## Supplemental Data

**Files in this Data Supplement:**

- Supplimental figures and a table - Supplimental Figures and a Table
