# Supplementary material for: Macrophage deficiency of Akt2 reduces atherosclerosis in Ldlr null mice
Source: J Lipid Res. 2014 Nov;55(11):2296–308. doi: 10.1194/jlr.M050633 (PMC4617132; doi:10.1194/jlr.M050633)
Supplement: Supplemental Data [file supp_M050633_jlr.M050633-1.pdf]

Babaev et al.

SUPPLEMENTAL DATA

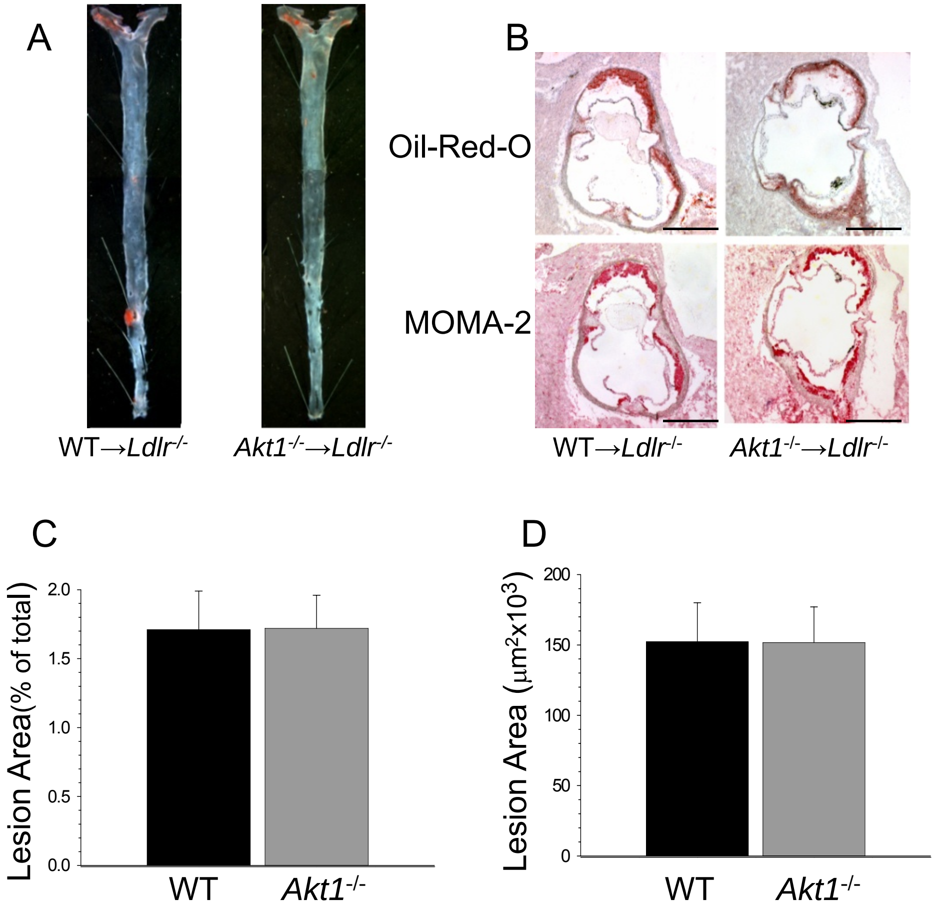

Figure S1**. Hematopoietic *Akt1* deficiency has no impact on early atherosclerosis in male *Ldlr$^{-/-}$* mice**. (**A,B**) Atherosclerotic lesions in the distal (A) and proximal (B) aorta of mice reconstituted with WT (n=9) or *Akt1$^{-/-}$* (n=11) FLC. Aortas were pinned out *en face* and stained with Sudan IV (A); serial sections were stained with Oil-Red-O or MOMA-2. Scale bars, 200 μm; a pin size, 10 mm. (**C,D**) The extent of atherosclerotic lesions in the distal and proximal aortas of *Ldlr$^{-/-}$* mice reconstituted with WT(■) or *Akt1$^{-/-}$*(■) FLC.

SUPPLEMENTAL DATA

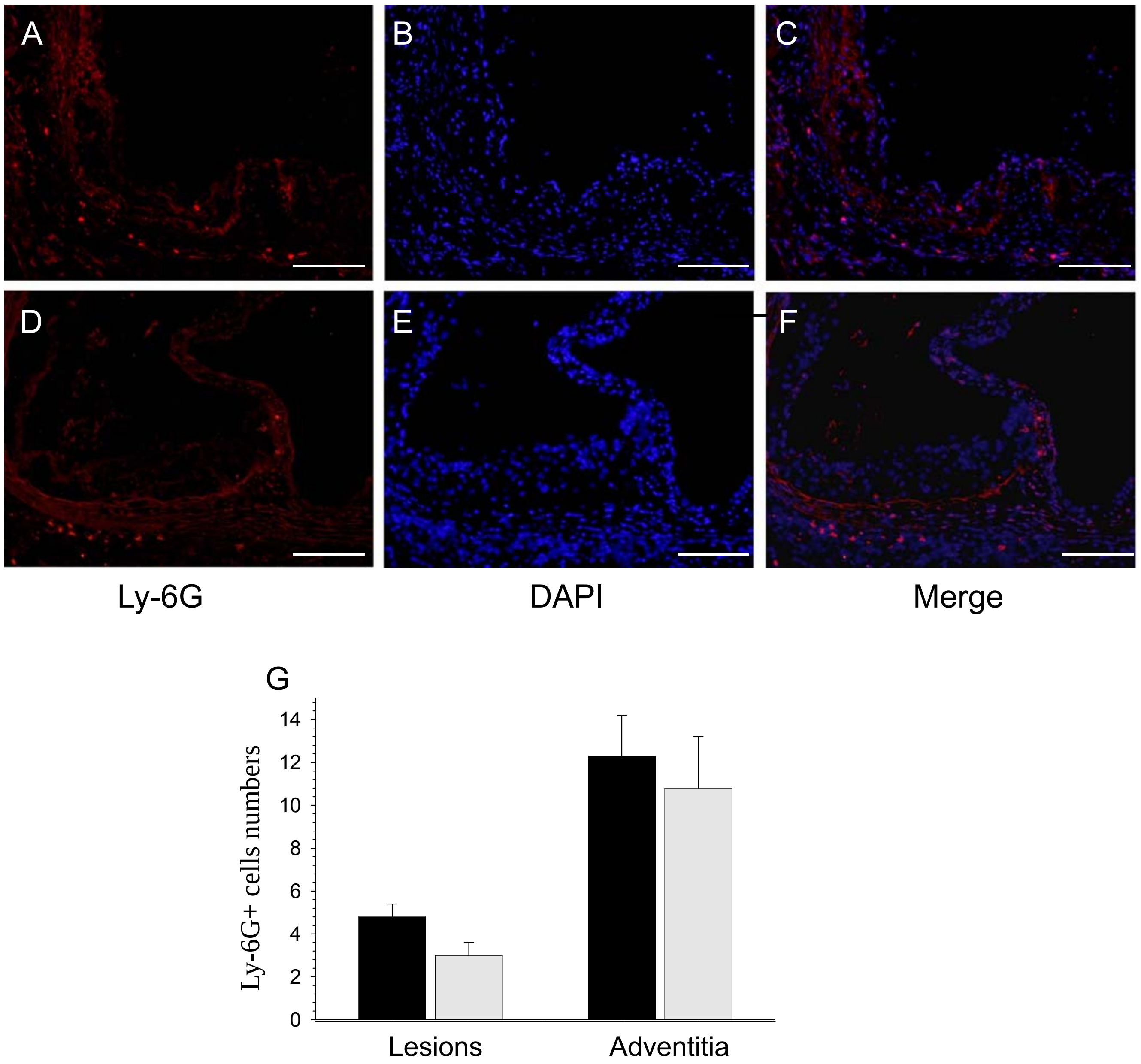

Figure S2**. Fluorescent staining of neutrophils in atherosclerotic lesions of *Ldlr*$^{-/-}$ mice transplanted with WT (A-C) or *Akt2*$^{-/-}$ (D-F) FLC.** (**A-F**) 5-micron sections of the aortic sinus were fixed with acetone and stained with the rat anti-mouse antibody to Ly-6G (1A8 clone; BioLegend) followed by Alexa Flour 594 goat anti-rat antibodies. Scale bars, 100 μm. (**G**) Percent of Ly-6G-positive cells in atherosclerotic lesions and aortic adventitia of WT→*Ldlr* $^{-/-}$ (■) and *Akt2*$^{-/-}$→*Ldlr*$^{-/-}$(■) mice after 8 weeks of the Western diet.

SUPPLEMENTAL DATA

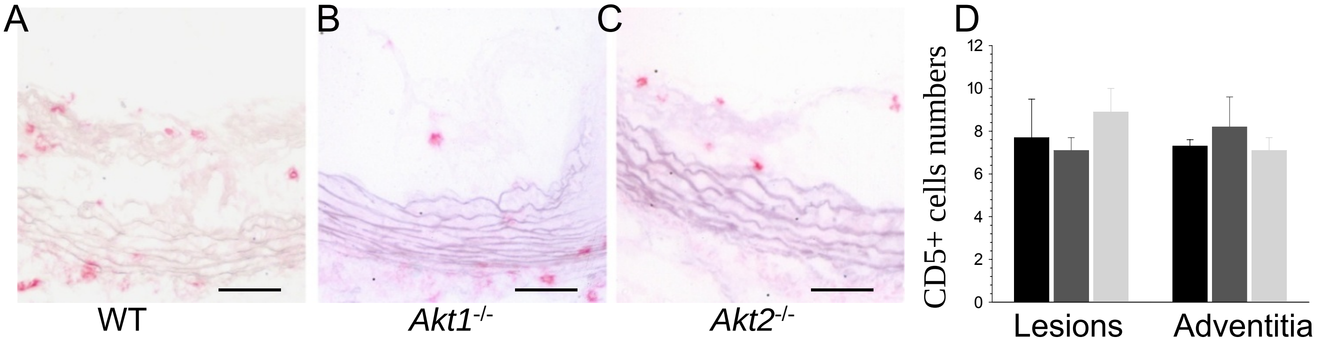

Figure S3**. CD5-positive cells in atherosclerotic lesions and aortic adventitia of *Ldlr*$^{-/-}$ mice reconstituted with WT, *Akt1*$^{-/-}$ and *Akt2*$^{-/-}$ FLC.** (**A-C**) 5-micron sections of the proximal aorta were fixed with acetone and stained with the rat anti-mouse Abs to CD5 (Ly1.2; PharMingen) followed by avidin-biotin complex labeled with alkaline phosphatase. Scale bars, 50 μm.(**D**) Percent of CD5-positive cells in atherosclerotic lesions and adventitia of WT→*Ldlr*$^{-/-}$(■), *Akt1*$^{-/-}$→*Ldlr*$^{-/-}$(■) and *Akt2*$^{-/-}$→*Ldlr*$^{-/-}$(■) mice fed with the Western diet for 16 weeks. .

SUPPLEMENTAL DATA

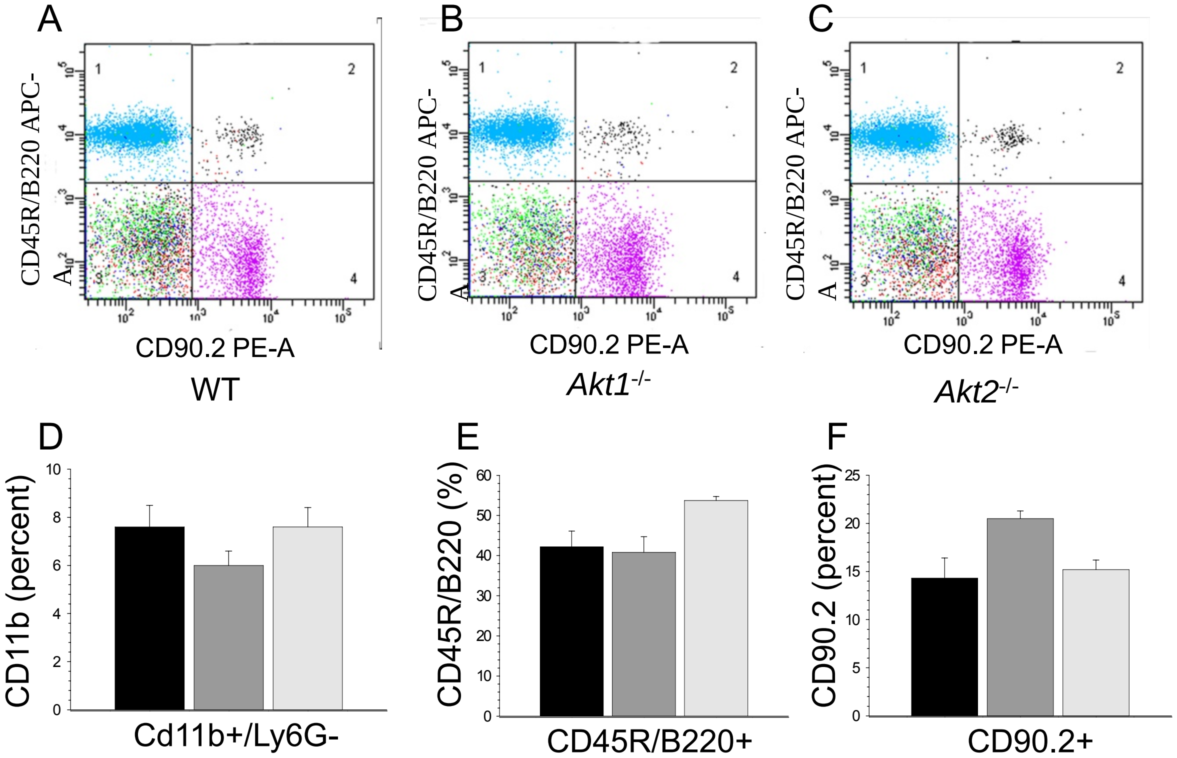

Figure S4**. Flow cytometry analysis of blood monocyes, B- and T-cells in *Ldlr*$^{-/-}$ mice transplanted with WT, *Akt1*$^{-/-}$ and *Akt2*$^{-/-}$ FLC.** (**A-C**) Analysis of the blood cell surface markers CD45R/B220 (blue color) and CD90.2 (violet color) in WT (A), *Akt1*$^{-/-}$ (B) and *Akt2*$^{-/-}$ (C) mice. Blood was isolated by retro-orbital bleeding, erythrocytes were hemolyzed by the BD lysing buffer and cells were analyzed by multicolor flow cytometry using Abs to CD11b FITC, CD45R/B220 APC and CD90.2 PE. (**D-F**) Percent of CD11b+/Ly6G- monocytes (D), CD45R/B220+ (E) and CD90.2+ (F) blood cells in recipient mice reconstituted with WT (■), *Akt1*$^{-/-}$(■) and *Akt2*$^{-/-}$(■) FLC**.**

SUPPLEMENTAL DATA

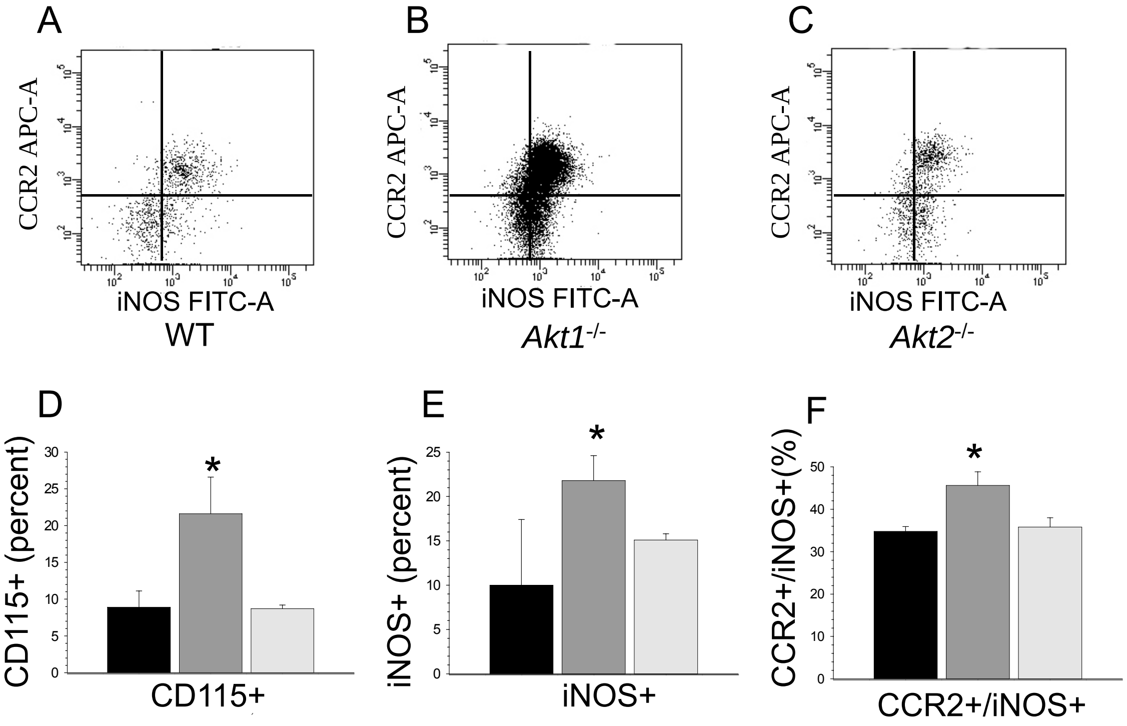

Figure S5**. CD115, iNOS and CCR2 expression in monocytes isolated from *Ldlr*$^{-/-}$ mice reconstituted with WT, *Akt1*$^{-/-}$ and *Akt2*$^{-/-}$ FLC on a chow diet.** (**A-D**) CD115-gaited flow cytometry analysis of CCR2 and iNOS expression in blood monocytes isolated from mice with WT (A), *Akt1*$^{-/-}$(B) and *Akt2*$^{-/-}$(C) FLC. (**D-F**) Percent of CD115+, iNOS+ and iNOS+/CCR2+ monocytes in *Ldlr*$^{-/-}$ mice reconstituted with WT(■), *Akt1*$^{-/-}$(■) and *Akt2*$^{-/-}$(■) FLC (*p<0.05 by One Way Analysis of Variance, Tukey test).

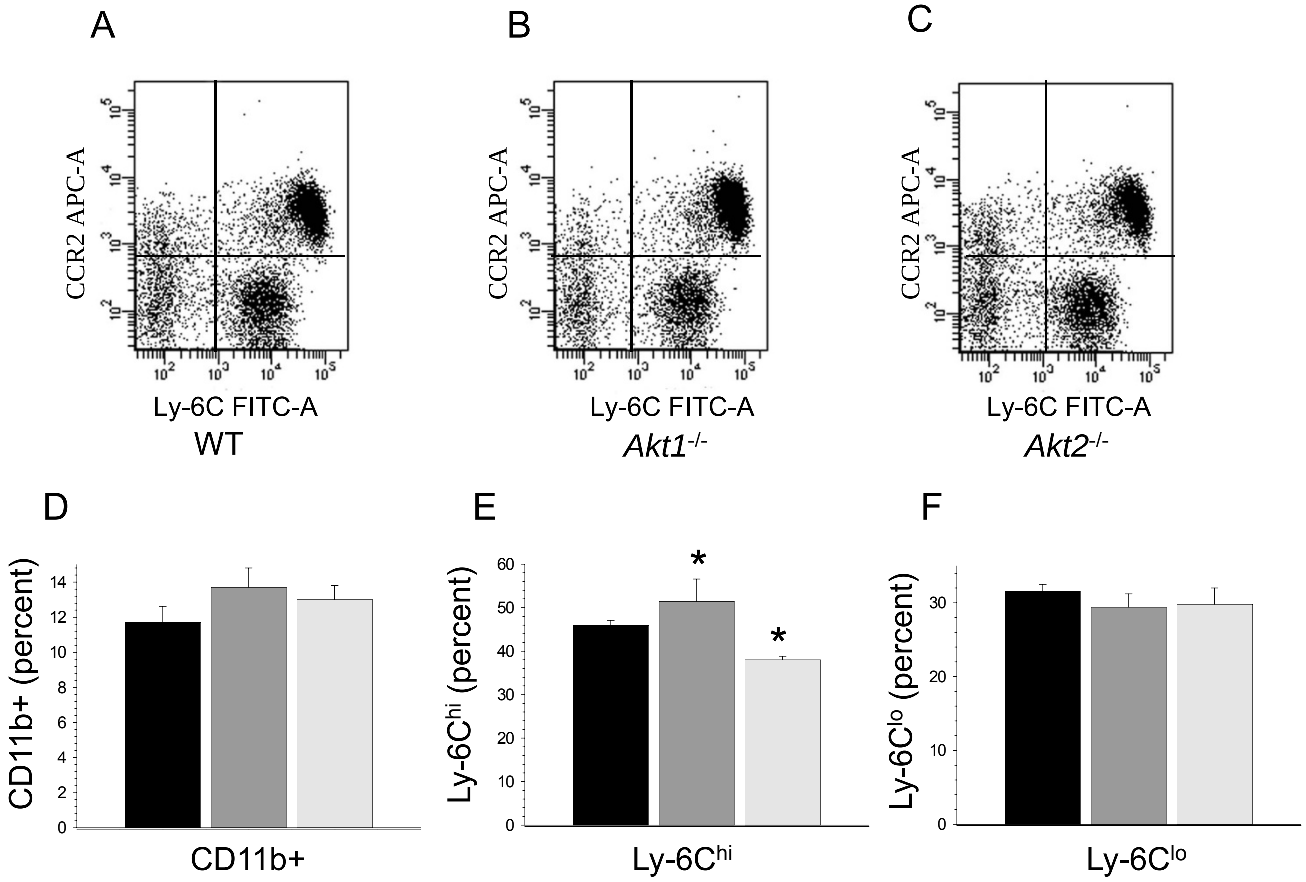

Figure S6**. CD11b, Ly-6C^{hi} and Ly-6C^{lo} expression in monocytes isolated from *Ldlr$^{-/-}$* mice reconstituted with WT, *Akt1$^{-/-}$* and *Akt2$^{-/-}$* FLC after 6 weeks of Western diet.** (**A-D**) CD11b-gaited flow cytometry analysis of Ly-6C in blood monocytes isolated from mice with WT (A), *Akt1$^{-/-}$*(B) and *Akt2$^{-/-}$*(C) FLC. (**D-F**) Percent of CD11b+, Ly-6C^{hi} and Ly-6C^{lo} monocytes from *Ldlr$^{-/-}$* mice reconstituted with WT(■), *Akt1$^{-/-}$*(■) and *Akt2$^{-/-}$*(■) FLC (*p<0.05 by One Way Analysis of Variance, Tukey test).

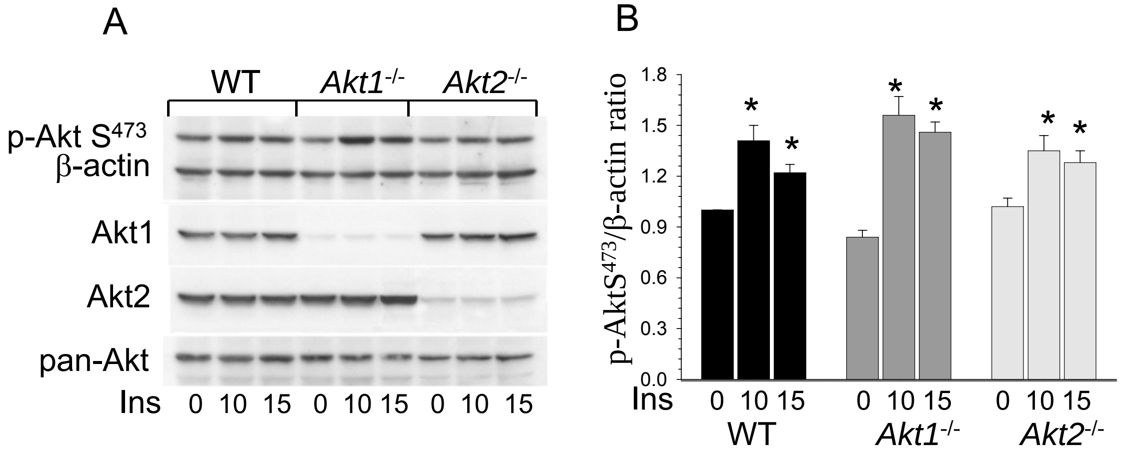

Figure S7**. Akt signaling in WT(■),** ***Akt1***$^{-/-}$**(■) and** ***Akt2***$^{-/-}$**(■) cells in response to insulin.** (**A**) WT, *Akt1*$^{-/-}$ and *Akt2*$^{-/-}$ peritoneal macrophages were untreated or treated with insulin (100nM) for 10 or 15 min. Proteins were extracted, resolved and analyzed by Western blot. (**B**) The ratio of p-Akt/b-actin is presented as the average (mean+SEM) of three different experiments (*p <0.05 compared to untreated WT cells by One Way Analysis of Variance).

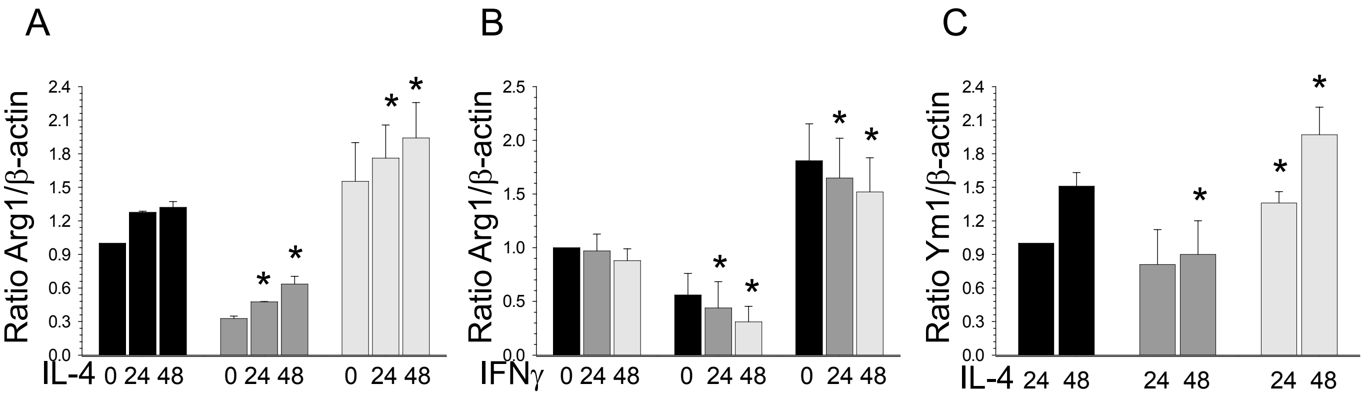

Figure S8. **Priming WT, *Akt1*$^{-/-}$ and *Akt2*$^{-/-}$ macrophages to M1 or M2 phenotype.** WT (■), Akt1$^{-/-}$(■) and Akt2$^{-/-}$(■) peritoneal macrophages were untreated (0) or treated with IL-4 (A,C) or IFNγ (B) for 24 and 48 hours; Graphs represent data of the Western blot analysis (Fig 4); *p<0.05 compared to control WT cells at the same time point by One Way Analysis of Variance, Tukey test.

Table S1. **LPS-altered and *Akt2*-dependent miRNAs in peritoneal macrophages from WT mice**

miRNA	Fold Change	P-Value
mmu-miR-1982.2	108.06	0.0455
mmu-miR-715	3.89	0.0293
mmu-miR-1896	3.23	0.0269
hsa-miR-151-5p	2.36	0.0039
rno-miR-196c	2.36	0.0072
mmu-miR-18a-3p	2.31	0.0109
mmu-miR-674-3p	2.27	0.0408
mmu-miR-362-5p	2.10	0.0189
hsa-miR-125a-5p	1.83	0.0087
hsa-miR-126-5p	1.73	0.0145
hsa-miR-221	1.64	0.0493
hsa-miR-146b	1.60	0.0454
hsa-miR-338-3p	1.55	0.0154
hsa-miR-222	1.51	0.0014
mmu-miR-1941-3p	-2.90	0.0393
mmu-miR-1938	-3.00	0.0235
mmu-miR-31-3p	-3.60	0.0281
